# Investigation of the myelin-amyloid interplay in Alzheimer’s disease: insights from novel dsCMA imaging in mouse and human brains

**DOI:** 10.1186/s40478-026-02317-8

**Published:** 2026-05-14

**Authors:** Hanpeng Xu, Sumit Nanda, Jiandong Sun, Mitchell Rudd, Lin Gou, Haley Marks, Christopher K. Williams, Shino Magaki, Harry V. Vinters, Houri Hintiryan, Hong-Wei Dong

**Affiliations:** 1 Brain Research and Artificial Intelligence Nexus (B.R.A.I.N.), Department of Neurobiology, David Geffen School of Medicine, University of California Los Angeles, Los Angeles, CA USA; 2 California NanoSystems Institute (CNSI), University of California Los Angeles, Los Angeles, CA USA; 3 Department of UCLA Pathology and Laboratory Medicine, Ronald Reagan UCLA Medical Center, University of California Los Angeles, Los Angeles, CA USA

## Abstract

Myelin deterioration impairs neural communication and may contribute to neurodegenerative diseases such as Alzheimer’s disease (AD). However, the spatiotemporal relationship between myelin degeneration and AD pathology remains unclear. We developed a novel imaging method—double-scan Cyto- and myelo-architecture (dsCMA)—that integrates multi-fluorescent labeling of cytoarchitecture and pathological markers with brightfield myelin staining on the same tissue sections. Coupled with computational tools for automated detection and quantification of amyloid-beta (Aβ) plaques and myelinated axons, dsCMA enables systematic analysis of Aβ–myelin interactions in 5xFAD mice and postmortem human AD tissues. In mice, we identified progressive, region-specific myelin disruption associated with Aβ accumulation, particularly in the hippocampus and cortex. Human samples revealed variable Aβ–myelin spatial relationships across individuals and regions, with evidence supporting a potential myelin origin for some plaques. These findings offer new insights into Aβ–myelin interplay and establish dsCMA as a scalable platform for histopathological analysis in translational and longitudinal AD research.

**Supplementary Information:**

The online version contains supplementary material available at 10.1186/s40478-026-02317-8.

## Introduction

Myelinated neural fiber assemblies are essential for brain structure, enabling efficient electrical impulse conduction via the lipid-rich myelin sheath [1]. Oligodendrocytes not only insulate but also provide nutritional and structural support, optimizing neural circuit coordination critical for cognitive functions [2, 3]. White matter myelin bundles play a pivotal role in neuroimaging, offering insights into physiological and pathological states and influencing diagnostic and therapeutic strategies in neurology and psychiatry [4].

Alzheimer’s disease (AD), a progressive neurodegenerative disorder, is seen in two primary forms: early-onset familial and the more common late-onset sporadic AD. While traditional research focuses on gray matter disruptions, significant myelin contributions within these regions are often overlooked, despite their known importance [5, 6]. Myelin deterioration is suggested to impair neural communication and increase susceptibility to AD, though it remains debated whether myelin degeneration precedes or results from AD [7]. Regardless, myelin abnormalities are known to disrupt neural network functions, contributing to neurological decline [8].

Gray matter, which hosts neuron cell bodies and facilitates neural processing, is also impacted by myelin anomalies that could significantly affect AD progression and exacerbate disease effects [9–11]. However, traditional studies on brain-wide myelin defects have been hindered by challenges like low contrast and high costs associated with histological or immunohistochemical techniques. Such studies typically rely on selective tissue sampling and lack comprehensive or longitudinal analysis. Additionally, current visualization methods struggle to clearly depict the relationship between myelin changes and AD pathology, limiting our understanding of their interplay [12–15].

Given the complex and multifactorial nature of myelin–axon interactions in AD, we hypothesized that myelin alterations occur in a spatially and temporally dependent manner in relation to Aβ plaque formation. We further reasoned that detailed morphological characterization of these interactions could provide a foundation for generating new mechanistic hypotheses regarding Aβ-associated myelin pathology.

In this study, we introduce an optimized myelin staining method combined with advanced imaging technology called double scans for Cyto- and myelo-architecture (dsCMA) to visualize myelinated fibers within gray and white matter in both mouse and human brains with exceptional details. We applied this technique to the 5xFAD mouse model of early-onset AD and post-mortem human AD specimens, focusing on (1) amyloid-myelin morphology correlations and (2) their spatial relationship across different stages of the accumulation of amyloid plaques. Our findings uncover a nuanced spatial relationship between amyloid-beta deposition and myelin morphology within the 5xFAD model, evident from as early as 3 months of age and persisting into later stages. Regional variations in myelin response to amyloid accumulation were observed, with some areas showing resilience and others manifesting changes independent of amyloid presence, a pattern also consistent in human AD samples. We also developed a novel quantitative method to examine the complex spatial and temporal interactions between amyloid plaques and myelin integrity, focusing on the dorsal and ventral hippocampus and the retrosplenial cortex. Our findings highlight the need for further research to elucidate these interactions and their implications for AD neuropathology and neural network function.

It is important to note that the etiology and pathological progression of AD are multifactorial, involving multiple converging mechanisms. Beyond Aβ deposition, tau pathology represents a central driver of neurodegeneration and cognitive decline in both typical and atypical dementias. The accumulation of hyperphosphorylated tau disrupts cytoskeletal integrity, impairs axonal transport, and contributes to synaptic dysfunction [12–14]. These tau-related changes likely act in concert with Aβ toxicity, glial activation, and myelin disruption, collectively shaping the spatiotemporal heterogeneity of AD pathology. In the future, our approach can be extended to investigate the spatial and temporal relationships between myelin alterations and tauopathies, another key dimension of AD pathogenesis.

## Results

### Heterogenous spatial relationships coexisted between Abeta deposits and myelinated axons in 5xFAD mice under ScoRe and confocal microscopy

We first explored the spatial interactions between Aβ deposits and myelinated fibers by Spectral Confocal Reflectance (SCoRe) imaging. Leveraging the reflective properties of compact myelin, SCoRe imaging provided a sensitive, label-free delineation of myelin structures, and minimized manipulation artifacts.

**Fig. 1:**
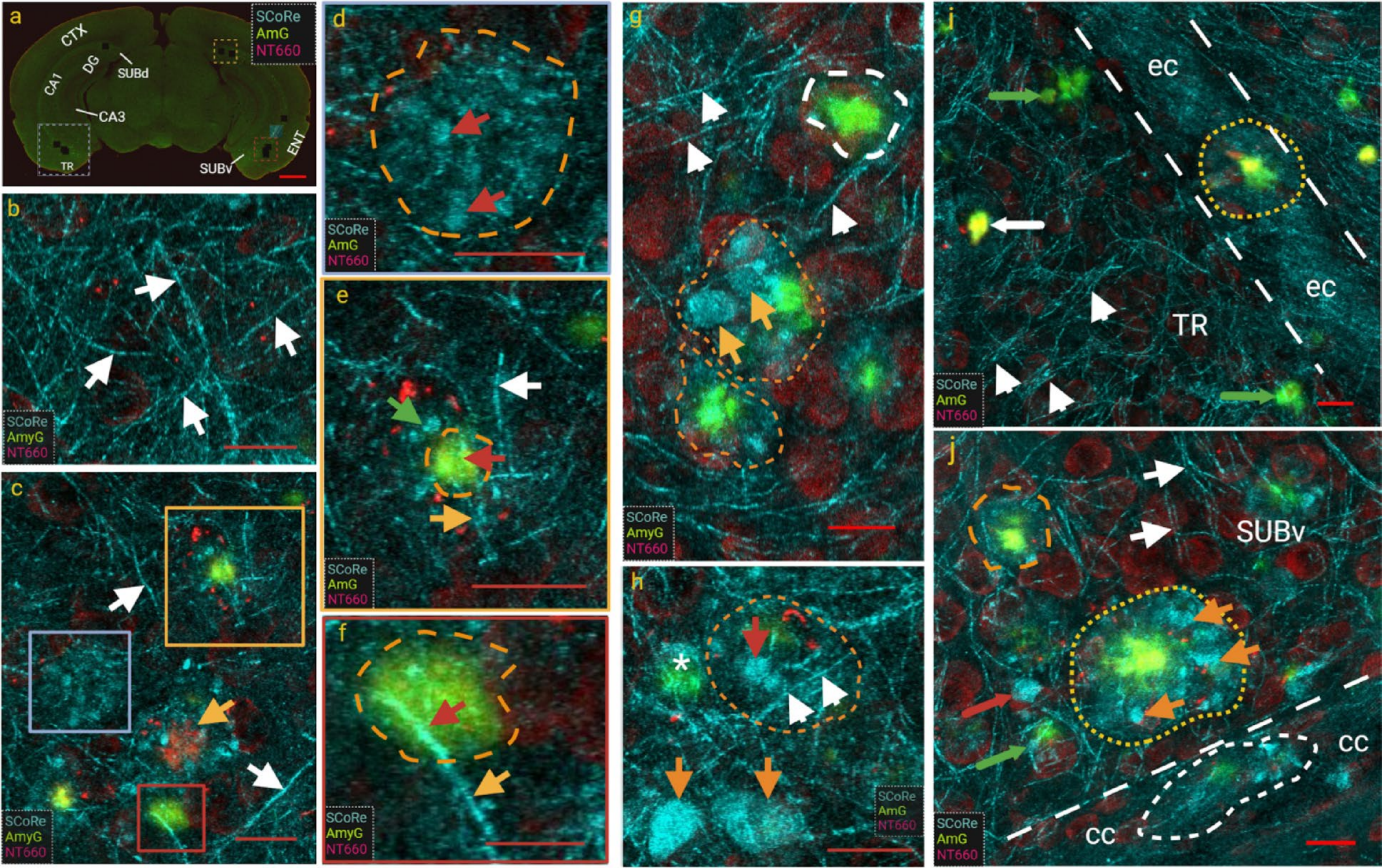
Spectral confocal reflectance (SCoRe) and fluorescence confocal imaging reveal heterogeneous myelin morphologies associated with amyloid-β (Aβ) deposits in 3-month-old 5xFAD male mouse brain. **a** Representative distribution of Aβ deposits (green) across cortex (CTX), ventral and dorsal subiculum (SUBv, SUBd), entorhinal cortex (ENT), post-piriform transition area (TR), dentate gyrus (DG), and hippocampal CA1 and CA3. Scale bar: 1 mm. **b** Enlarged view of the region indicated in **a**, showing normal compact myelinated fibers (SCoRe signal, pseudo-colored cyan; arrows) with multiple trajectories. Scale bar: 20 µm. **c** Enlarged view of the yellow dashed box in **a,** showing coexisting Aβ deposits (green), myelinated fibers (cyan), dystrophic neurite spheroids (cyan), and degenerating cells (red). Scale bar: 20 µm. **d** Higher-magnification view of the cyan box in **c,** illustrating clustered dystrophic neurite spheroids (arrows) with high signal density, obscuring individual spheroid boundaries. Scale bar: 20 µm. **e** Enlarged view of the orange box in **c,** showing dense-core Aβ deposit traversed by a myelinated fiber (arrow), with additional nearby normal-appearing myelinated fibers and dystrophic swellings. Scale bar: 20 µm. **f** Dense-core Aβ deposit lacking surrounding dystrophic neurites, with a myelinated fiber passing through the plaque core without obvious morphological disruption. Scale bar: 20 µm. **g** Enlarged view of the yellow dashed box from image **a,** showing representative examples of dense-core halo Aβ deposits with (central orange dashed circles) or without (white and low orange dashed circles) associated dystrophic neurite spheroids. Scale bar: 20 µm. **h** Enlarged view of the red box in **a.** Diffuse Aβ deposit containing both normal-appearing myelinated fibers and dystrophic neurite spheroids, including large spheroids occurring outside detectable Aβ deposits. Scale bar: 20 µm. **i** Enlarged view of the white dashed box in **a.** Dense-core halo Aβ deposit within white matter (double dashed lines), accompanied by local signal abnormalities and intermingled myelinated fibers, without detectable dystrophic spheroids. Abbreviations: ec, external capsule; TR, post-piriform transition area. Scale bar: 20 µm. **j** Enlarged view of the red dashed box in **a.** Large dense-core halo Aβ deposit associated with multiple dystrophic neurite spheroids, contrasted with an adjacent deposit lacking spheroids. A diffuse white matter Aβ deposit shows local alterations of myelin bundle organization. Abbreviations: cc, corpus callosum; SUBv, ventral subiculum. Scale bar: 20 µm

Using the Aβ-specific fluorescent dye Amylo-Glo (AmG), we observed heterogeneous Aβ deposit (plaque) morphologies, ranging from diffuse to compact, in the forebrain of 3-month-old 5xFAD male mice. The deposits were most abundant in the subiculum, hippocampus, and entorhinal cortex, while appearing sparser and more scattered in the neocortex (Fig. 1a).

Under SCoRe, normal myelinated axons were identified as consistently calibrated, slender strands (Fig. 1b), dystrophic neurites were identified by their morphologies by their various sizes (swelling < 3 μm and spheroid > 3 μm). (Fig. 1c).

Aβ deposits and myelinated fibers showed various spatial relationships, three types of relationship were categorized.

Type I: Dystrophic neurite clusters devoid of Aβ deposits, with configurations that obscured individual neurite profiles (Fig. 1d, blue box in Fig. 1c). In this type, dystrophic neurites were sometimes independent of Aβ deposits, occasionally co-localizing with myelinated fibers in diffuse Aβ areas (Fig. 1h).

Type II: Dystrophic and normal myelinated fibers interlaced with dense-core or dense-core halo Aβ deposits, some fibers penetrated the deposits (Fig. 1e, yellow box in Fig. 1c). In this type, the dimensions of dystrophic neurites bore distinct correlations with the Aβ deposits’ size (Fig. 1g).

Type III: Normal myelinated fibers penetrated cross dense core Aβ deposits without unaltered trajectory (Fig. 1f, red box in Fig. 1c).

Notably, at 3 months, very few Aβ deposits were found in white matter at three months, with myelin-like signal alterations near Aβ deposits (Fig. 1i, j), and numerous dystrophic neurite-like signals resembling a typical floral pattern were found in the hippocampus (Fig. 1j).

**Fig. 2:**
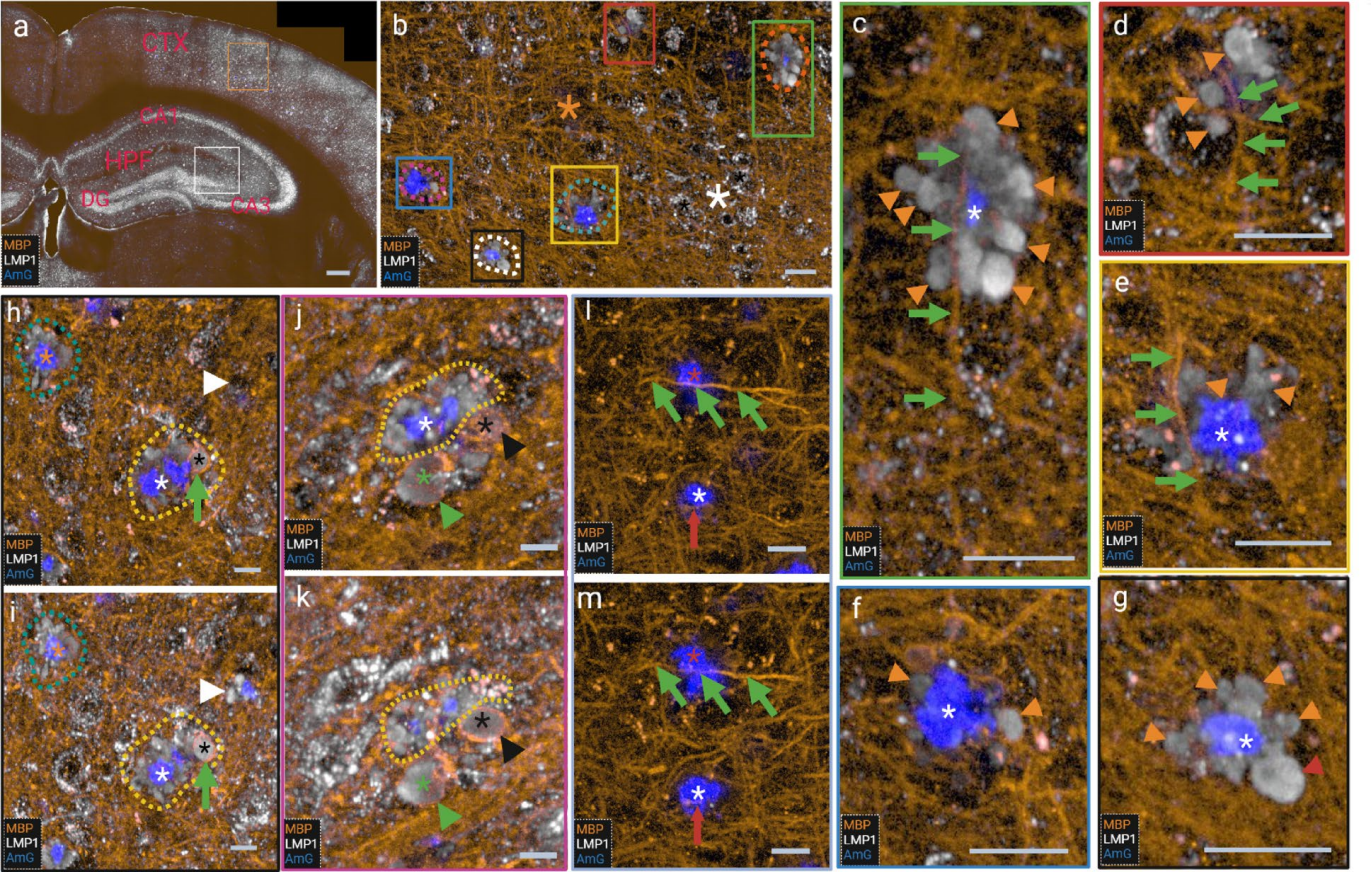
Immunofluorescence confocal imaging reveals heterogeneous spatial relationships among Aβ deposits, dystrophic neurites, and myelinated fibers in 3-month-old 5xFAD mouse brain. a Representative image from a 3-month-old male 5xFAD mouse showing Aβ deposits (blue), LAMP1+ dystrophic neurites (white), and MBP+ myelinated fibers (orange). Boxes indicate hippocampal (white) and neocortical (orange) regions shown at higher magnification. Scale bar: 200 µm. **b** Enlarged view of the hippocampal region (white box in **a**) showing multiple myelinated fibers with distinct trajectories (orange star), grain-like LAMP1+ vesicles within neuronal cell bodies (white star), and diverse plaque-associated phenotypes. A small Aβ deposit interlaced with LAMP1+ dystrophic spheroid clusters is shown (green box; dashed orange circle). LAMP1+ spheroids without detectable Aβ deposits are indicated (red box). An Aβ deposit ringed by dystrophic neurites and spheroids is shown (yellow box; dashed cyan circle). A large Aβ deposit with scant dystrophic neurites is indicated (blue box; dashed red circle). A small Aβ deposit associated with multiple spheroids is also shown (black box; dashed white circle). Scale bar: 30 µm. **c** Enlarged view of the green box in **b**, showing a small Aβ deposit (white star) encircled by multiple dystrophic neurite spheroids (orange arrowheads). A normal-appearing myelinated fiber traverses the center of the deposit without detectable abnormality (green arrows). Scale bar: 30 µm. **d** Enlarged view of the red box in **b**, showing multiple dystrophic neurite spheroids (orange arrowheads) and normal-appearing myelinated fibers (green arrows) forming a cluster without an associated Aβ deposit. Scale bar: 30 µm. **e** Enlarged view of the yellow box in **b**, showing dystrophic neurite spheroids (orange arrowheads) and a normal-appearing myelinated fiber (green arrow) colocalized with a large Aβ deposit (white star). Scale bar: 30 µm. **f** Enlarged view of the blue box in **b**, showing a large Aβ deposit (white star) associated with two dystrophic neurite spheroids (orange arrowheads). Scale bar: 30 µm. **g** Enlarged view of the black box in **b**, showing multiple small dystrophic neurite spheroids (orange arrowheads) and a large spheroid (red arrowhead) surrounding a small Aβ deposit (white star). Scale bar: 30 µm. **h, i** Serial optical sections illustrating morphological variation of dystrophic neurite spheroids across z-levels around the same Aβ deposit (dashed yellow circle). One large spheroid is enveloped by MBP signal (black star; green arrow). Changes in Aβ deposit morphology (white star) and associated spheroids are observed across optical levels. An adjacent Aβ deposit (orange star) exhibits corresponding changes in plaque-associated spheroids and normal-appearing myelinated fibers (dashed cyan circle), including spheroids appearing at different z-levels but occupying the same spatial position (white triangle). Section interval: 2 µm. Scale bar: 20 µm. **j, k** Serial optical sections of an Aβ deposit (white star) showing z-dependent changes in surrounding dystrophic neurite spheroids (dashed yellow circle). MBP signal (triangle arrows) partially encircles one large LAMP1+ spheroid (green star) and completely encircles another (black star), with MBP+ spheroids appearing independent of the Aβ deposit. Section interval: 3 µm. Scale bar: 20 µm. **l, m** Enlarged view of the neocortical region (yellow box in **a**) showing serial optical sections in which a normal-appearing myelinated fiber (green arrows) traverses the dense core of an Aβ deposit (red star). A second Aβ deposit (white star) is partially associated with a newly emerging myelinated fiber (red arrows). No dystrophic neurite spheroids are associated with these deposits. Section interval: 2 µm. Scale bar: 20 µm

To further explore their molecular properties and spatial relationship of the morphological signals observed under SCoRe, conventional confocal imaging and 3D reconstructions were used with myelin specific marker (myelin-basic protein, MBP) and dystrophic neurites specific marker( lysosomal-associated membrane protein 1, LAMP1) staining (Fig. 2a).

Under conventional confocal microscopy, MBP positive myelinated fibers with variable trajectories intertwined with LAMP1-positive signals in neuronal soma and fibers (Fig. 2b). Similar to ScoRe, various spatial relationships of Aβ deposits and myelinated fibers were observed (Fig. 2b, color boxes).

Under high resolution, normal morphology MBP+ myelinated fibers were observed traversing the core of small and large AmG+Aβ deposits amidst LAMP1+/MBP- dystrophic neurite spheroids, without trajectories and integrity change (Fig. 2c, e). Local clusters of LAMP1+ dystrophic neuritic spheroids and swellings and MBP+ myelinated fibers were found independently of AmG+Aβ deposits (Fig. 2d). The proximity localization of normal morphology MBP+ myelinated fibers with LAMP1+ dystrophic neurite spheroids and AmG+Aβ deposits (Fig. 2f, g) indicated that Aβ deposits were not inherently impediment/toxic to myelin fibers, at least for some myelinated fibers.

To further differentiate the myriad forms of neuritic pathology, we used optical virtual slicing techniques to observe Aβ plaques and trace MBP+ signals along individual myelinated fibers at different levels. Our data showed that Aβ plaques exhibited irregular shapes along the Z-axis, with some regions protruding above adjacent tissue, and confirmed that MBP+/LAMP1+ myelinated dystrophic neurite spheroids could occur independently of Aβ aggregates (Fig. 2h, i and j, k, black asterisks).

To our surprise, normal morphology MBP+ myelinated fibers were observed traversing rightly through the dense-core AmG+Aβ plaques, maintaining their trajectories and integrity and lesion (Fig. 2l, m, arrowheads). Notably, these dense-core Aβ plaques had no associated LAMP1+ dystrophic neurite spheroids. Around 32.4% of plaques in the cortex and 19.3% in the hippocampus had intact MBP positive myelinated fibers passing through the dense core of Aβ plaques at 3 months. Further analysis showed that there were variations in myelination patterns of dystrophic neurites, from fully encased to partially ensheathed in gray and white matter in 3- and 7-month-old age (Extended Data Fig. 1).

To validate the specificity and sensitivity of Aβ deposits detection by AmG, we compared the staining outcomes of AmG and Thioflavin-S (ThioS) on the same sections. A sequential imaging of AmG and ThioS staining exhibited 98% overlap (1056 counted) in plaque localization and morphology (Extended Data Fig. 2).

We further compared the distribution of AmG+Aβ deposits with that of LAMP1, presenilin-1 (PSEN1, part of γ-secretase complex), and APP/Abeta (antibody NAB228) using specific antibodies on the same section (Extended Data Fig. 3a–h). Over 96% of AmG+plaques are colocalized with APP, PSEN and LAMP1, with AmG concentrated at plaque cores and encircled by peripheral APP/PSEN1/LAMP1, which had similar and considerable overlap with each other (see Extended Data Fig. 3h).

**Fig. 3:**
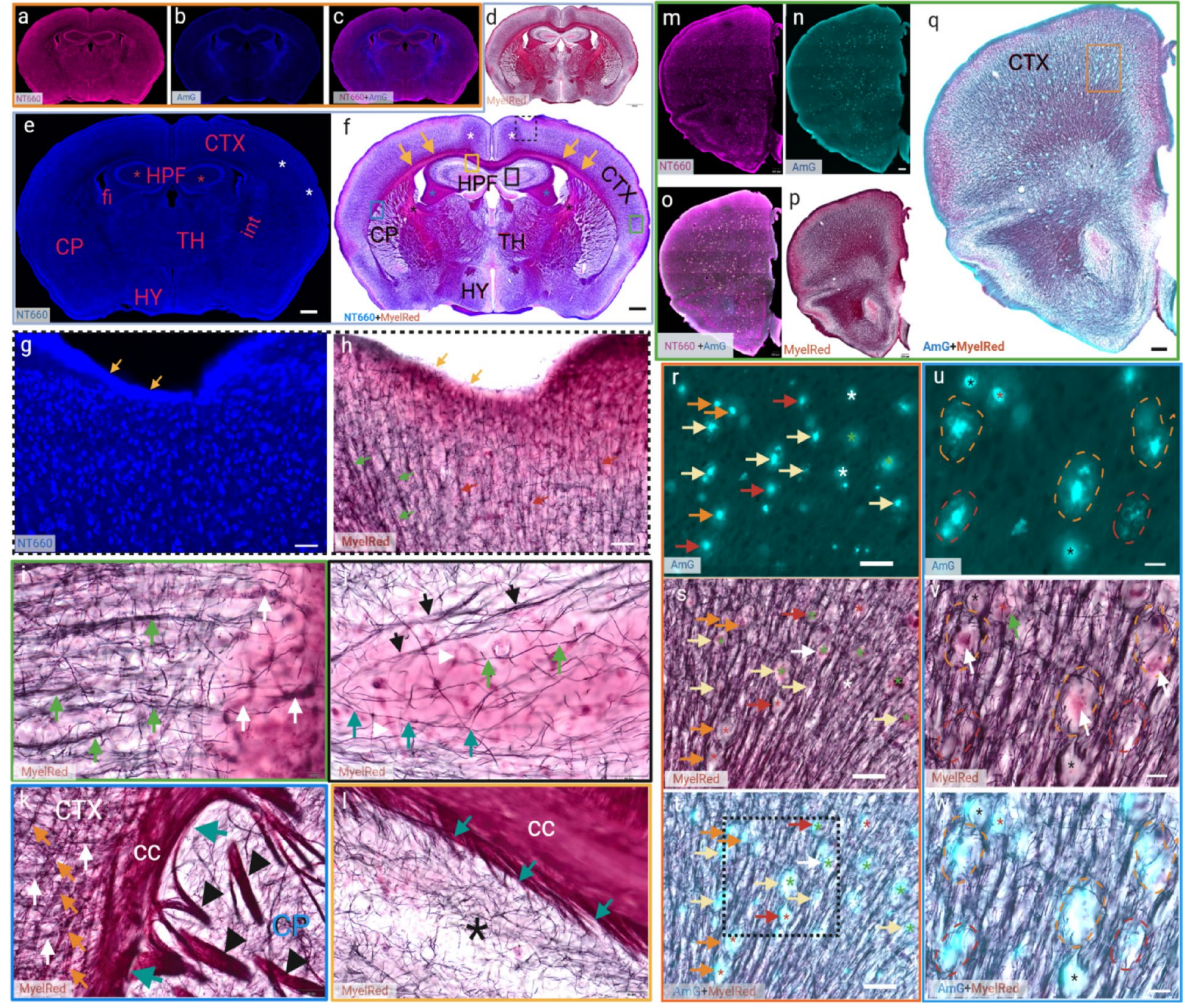
dsCMA pipeline enables integrated analysis of multiplex fluorescence signals and brightfield myelin microarchitecture from the same brain section in 3-month-old wild-type and 5xFAD mice. **a**–**f** dsCMA workflow in wild-type brain. **a** Nissl staining highlights cytoarchitecture. **b** Amylo-Glo (AmG) staining for Aβ deposits (no signal detected). **c** Overlay of **a** and **b**. **d** Brightfield MyelRed staining of the same section reveals myeloarchitecture, including individual myelinated fibers and bundle patterns. **e** Pseudo-colored Nissl image with cortex (white star) and hippocampus (red star) indicated. **f** Warpy co-registration of **d** and **e**, highlighting corpus callosum (orange arrows), fimbria (green star), cingulum (white star), and internal capsule (black star). Scale bars: **a**–**d**, 500 µm; **e, f**, 200 µm. **g**–**l** High-magnification views from **f** illustrating myelin microstructure. **g** Nissl staining of cortex and pial surface (orange arrows). **h** Corresponding MyelRed image showing densely packed radial fiber bundles (green arrows) and loosely organized fibers with variable trajectories (red arrows). **i** Enlarged view showing parallel bundles (green arrows), weakly stained blood vessels (white arrows), and intersecting myelinated fibers. **j** Enlarged view showing small bundles (black arrows), nodes of Ranvier (green/dark green arrows), and blood vessel profiles (white arrowheads), with relatively constant myelin segment diameter. **k** Enlarged view showing parallel bundles (white arrows) and tangential bundles (orange arrows) in deep cortical layers; boundary between corpus callosum (cc) and caudoputamen (CP) (blue arrows) and fiber bundles within CP (black arrowheads). **l** Enlarged view showing the white–gray matter boundary (blue arrows) and hippocampus (black star). Scale bar: 50 µm. **m**–**q** dsCMA workflow in 5xFAD brain. **m** Nissl staining. **n** AmG staining for Aβ deposits (pseudo-colored). **o** Overlay of **m** and **n**. **p** Brightfield MyelRed staining. **q** Warpy co-registration of **n** and **p**, enabling spatial mapping of Aβ deposits relative to myelinated fibers. Scale bar: 200 µm. **r**–**t** Enlarged view of the orange box in **p**. **r** AmG+ Aβ deposits with distinct morphologies, including diffuse plaques without dense core (white stars), weakly stained dense-core halo plaques (red arrows), dense-core halo plaques (orange arrows), and branched dense-core halo plaques (yellow arrows). **s** Corresponding MyelRed image showing diverse myelin abnormalities, including blank spots with central granular red–black staining blocks (green stars), blank spots without central staining blocks (red stars), altered bundle trajectories (orange arrows), disrupted bundles (orange arrows with green stars), and reduced bundle density (orange arrows only). **t** Registered overlay of **r** and **s** highlighting spatial relationships between plaque types and myelin defects. Scale bar: 75 µm. **u**–**w** Enlarged view of the black dashed box in **t**. **u** AmG+ plaques including round dense-core halo plaques (stars), large irregular branched plaques (orange dashed circles), diffuse plaques without dense core (right red dashed circle), and diffuse plaques with dense core and halo (left red dashed circle). **v** Corresponding MyelRed image showing myelin defects at plaque locations, including blank spots with trajectory disruption and central bull’s-eye–like staining blocks (orange dashed circles; white arrows), blank spots with disrupted trajectories but no bull’s-eye block (black stars), blank spots with residual fibers (red star), and plaques without detectable blank spots or trajectory disruption (red dashed circles). **w** Warpy-registered overlay of **u** and **v**, highlighting heterogeneous and non-uniform correspondence between plaque morphology and myelin abnormalities. Scale bar: 75 µm

In summary, using SCoRe and confocal microscopic imaging techniques on 5xFAD mice, our findings have shown that while Aβ deposits were often associated with dystrophic neurites, these dystrophic neurites could occur independently with no association with Aβ deposits. Furthermore, these dystrophic neurites could be myelinated to various degrees. On the other hand, normal morphology myelinated fibers could penetrate through the dense core of Aβ deposits while maintaining their trajectory and integrity. We also observed that myelin integrity within the neocortex was relatively more preserved compared to the hippocampus, with a higher proportion of Aβ plaques retaining traversing myelinated fibers. All these underscore the complex and dynamic nature of amyloid pathology interactions with myelin at single fiber and regional level.

### Development of the double scan cyto- and myelo-architecture (dsCMA) method

To overcome the limitations of SCoRe, confocal imaging, and conventional immunohistochemistry (including limited specificity, low contrast, and high cost), we developed a double-scan cyto- and myelo-architecture (dsCMA) pipeline (Extended Fig. 4). This approach integrates the Warpy imaging framework [16] to register multiplex fluorescence images (e.g., Nissl, Aβ) with brightfield myelin images acquired from the same tissue section, enabling simultaneous high-resolution analysis of cytoarchitecture and myeloarchitecture (Fig. 3a–f).

**Fig. 4:**
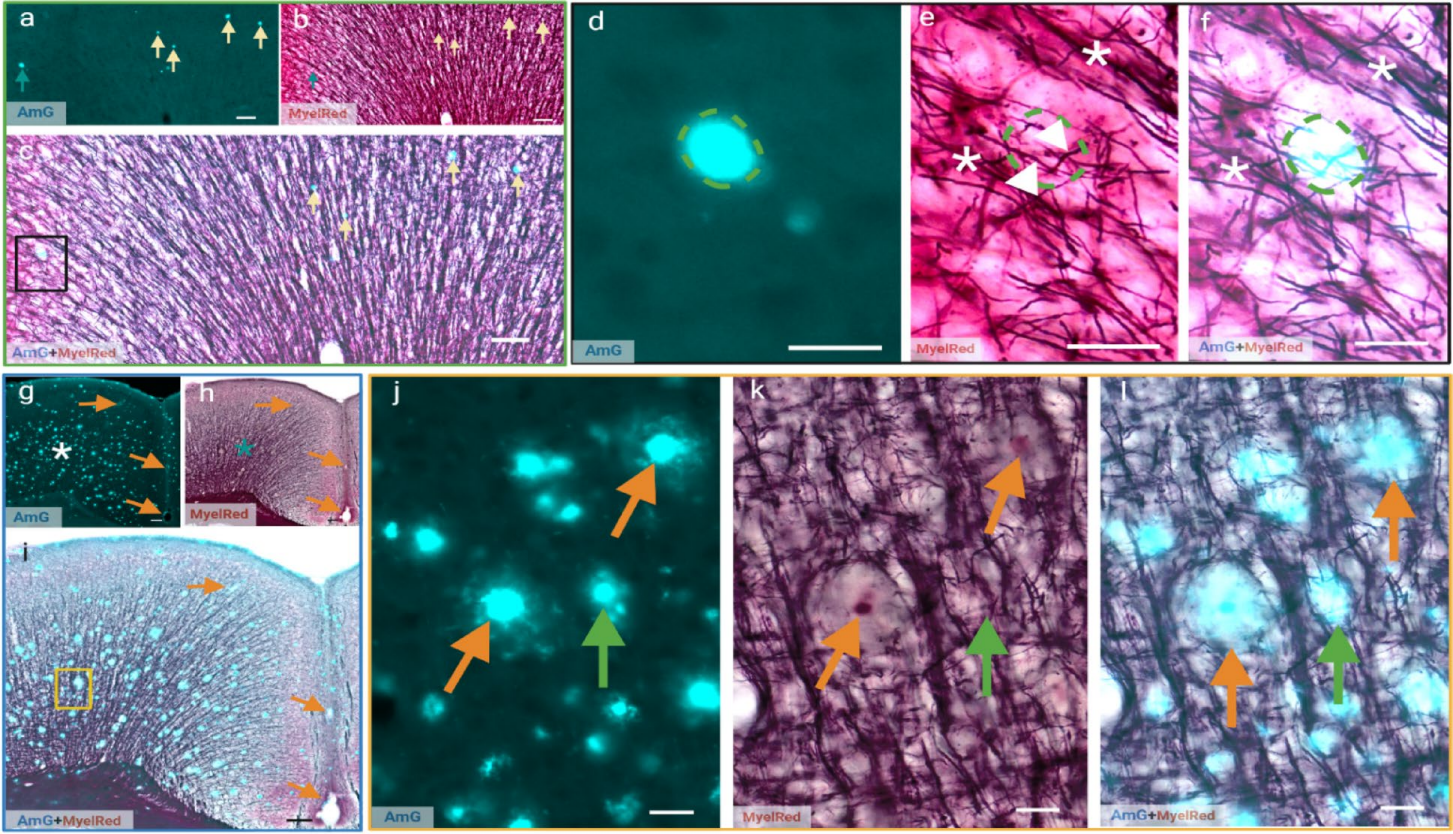
dsCMA pipeline reveals age-dependent spatial relationships between cortical amyloid-β (Aβ) deposits and myelinated fibers in 2- and 7-month-old 5xFAD mice. **a**–**c** In 2-month-old 5xFAD mice, cortical Aβ deposits are embedded within radial myelin fiber bundles and individual fibers (arrows) without detectable myelin defects. Scale bar: 75 µm. **d**–**f** Enlarged view of the black box in **c**, showing a dense-core Aβ deposit without halo (dashed green circle; green arrow in **a**) colocalized with normal-appearing myelinated fibers (white arrowheads) and intact parallel bundles (white stars), with no detectable abnormalities. Scale bar: 25 µm. **g**–**i** In 7-month-old 5xFAD mice, abundant Aβ deposits and blood vessel–like structures (orange arrows) are present in the anterior cingulate cortex (white star in **g**). **h** The same region shows radial myelinated fibers (green star) and blood vessel–like structures (orange arrows). **i** Warpy co-registration of **g** and **h** highlights spatial associations between Aβ deposits and myelin defects. Scale bar: 100 µm. **j**–**l** Enlarged view of the orange box in **i**. **j** Aβ deposits with diverse morphologies coexist within the same region, including irregular branched dense-core halo plaques (orange arrows), small dense-core halo plaques, and tiny deposits (green arrow). **k** Corresponding myelin image shows plaque-associated blank spots with disrupted fiber trajectories and central bull’s-eye–like staining blocks (orange arrows), whereas an adjacent Aβ deposit colocalizes with normal-appearing myelinated fibers (green arrow). **l** Warpy co-registration of **j** and **k** illustrates heterogeneous plaque–myelin spatial relationships. Scale bar: 25 µm

The dsCMA workflow progresses through a tri-phasic protocol:

1. *Tissue processing and initial imaging:* Brain sections were stained by lipid-keeping fluorescent methods to show cellular architecture (Nissl) or other normal or pathological changes (e.g., AmG+Aβ deposits), then imaged under fluorescent microscope (fluorescent, confocal or SCoRe microscopy) [17].
2. *Re-staining and second imaging:* After removing the coverslips, sections are re-stained with a gold phosphate-based solution to highlight myelin (myelRed) [18, 19] and imaged under brightfield to capture detailed myelin morphologies.
3. *Image integration:* The Warpy method [16] was employed to integrate images from step 1 and 2 for analyzing relationships of fluorescent (amyloid deposits) and brightfield (myelin) signals.

**Fig. 5:**
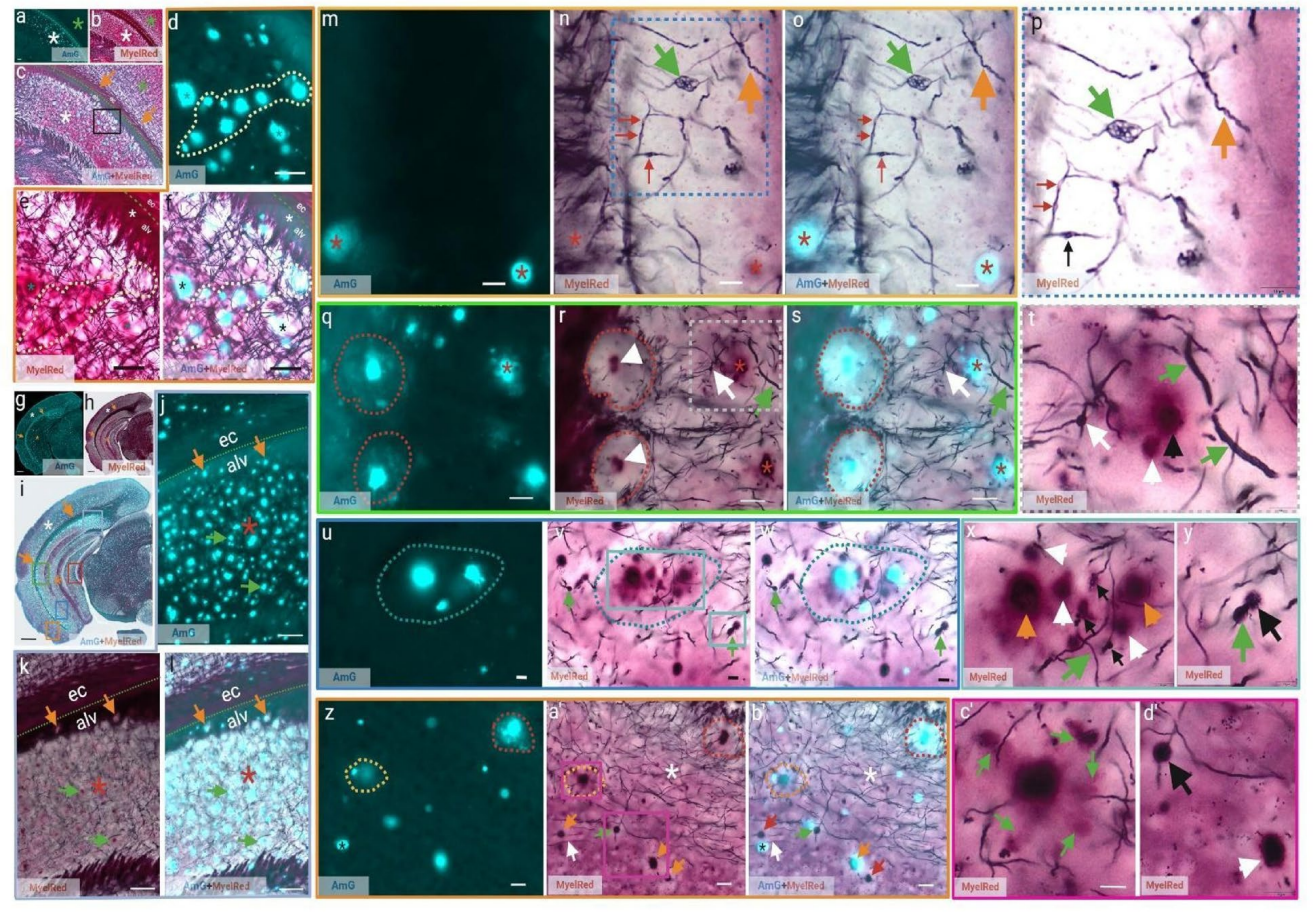
dsCMA pipeline reveals heterogeneous spatial relationships between hippocampal amyloid-β (Aβ) deposits and myelinated fibers in 2- and 7-month-old 5xFAD mice. **a**–**c** At 2 months, abundant Aβ deposits are present in the hippocampus (white star), with lower density in cortex (green star) and external capsule (orange arrows). Scale bar: 100 µm. **d**–**f** Enlarged view of the black box in **c**. **d** Individual Aβ deposits of varied size and morphology (stars) and clustered deposits (dashed yellow circle). **e** Corresponding myelin image shows plaque-associated blank spots with disrupted fiber trajectories (green stars) or minimal detectable abnormalities in gray matter (dashed yellow circle) and white matter (white star). **f** Warpy co-registration of **d** and **e**. Scale bar: 50 µm. **g**–**i** At 7 months, abundant Aβ deposits are observed in cortex (white star) and hippocampus (orange star), with sparse deposits in corpus callosum (orange arrows). Scale bar: 500 µm. **j**–**l** Enlarged view of the gray box in **i**. **j** Aβ deposits in subiculum (SUB; red star) are distributed in both gray matter (green arrows) and white matter (orange arrows). **k** Plaque-associated myelin abnormalities include central bull’s-eye–like staining blocks (green arrows) and disruption of parallel myelin bundles in white matter (orange arrows). **l** Co-registration of **j** and **k**. Scale bar: 100 µm. **m**–**p** Enlarged view of the orange box in **i**. **m** Two plaque morphologies are shown: loose-core halo plaque (left red star) and dense-core plaque without halo (right red star). **n** Diverse myelin morphologies are present, including tortuous fibers (orange arrow), network-like bundles (green arrow), normal fibers (red arrows), and dark spheroids (black arrow). **o** Co-registration of **m** and **n**. **p** Enlarged view of the blue dashed box in **o** highlights tortuous fibers (orange arrow), normal fibers (red arrows), and a spheroid (black arrow) in a region lacking detectable Aβ. Scale bar: 10 µm. **q**–**t** Enlarged view of the green box in **i**. **q** Aβ deposits are located at the gray–white matter boundary (dashed red circles) and within gray matter (red star). **r** Corresponding myelin image shows plaque-associated defects in white matter (dashed red circles) with central bull’s-eye staining blocks. Additional features include staining blocks at plaque locations in gray (red stars) and white matter (triangle arrows), a spheroid (white arrow), and a thick myelinated fiber (green arrow). **s** Co-registration highlights plaque–myelin spatial relationships. **t** Enlarged view of the dashed gray box in **r** shows a myelin spheroid (white arrow), thick myelinated fiber segments (green arrows), and Aβ deposits associated with (white triangle arrow) or without (black triangle arrow) central staining blocks. Scale bar: 25 µm. **u**–**w** Enlarged view of the blue box in **i**. **u** Clustered dense-core halo Aβ deposits (cyan dashed circle). **v** Corresponding myelin image shows multiple staining blocks intermingled with normal myelinated fibers, including fibers with spheroids (green arrows). **w** Co-registration of **u** and **v**. Scale bar: 25 µm. **x, y** Enlarged view of the cyan box in **v**. **x** Aβ deposits colocalize with large staining blocks (orange triangle arrows), whereas smaller staining blocks occur without detectable Aβ (white triangle arrows). Myelin spheroids (black arrows) and normal fiber segments (green arrows) are indicated. **y** Enlarged view of the small cyan box in **v** shows two closely positioned spheroids with distinct morphologies (green and black arrows) in the absence of detectable Aβ. Scale bar: 20 µm. **z**–**b**′ Enlarged view of the orange box in **i**. **z** Distinct plaque morphologies include a small dense core with eccentric faint halo (orange circle), a large dense-core plaque with surrounding grains and halo (red dashed circle), and a dense-core plaque without halo (black star). **a**′ Corresponding myelin image shows a large staining block with blurred profile and faint radial arms (orange circle). The plaque in the red dashed circle is associated with a central bull’s-eye staining block and surrounding white matter myelin defects, with residual normal fibers. Normal myelinated fibers (white star), a single normal fiber (white arrow), round staining blocks (orange arrows), and a myelin-associated spheroid (green arrow) are indicated. **b**′ Co-registration shows partial overlap between Aβ deposits and staining block/spheroid abnormalities. **c**′,** d**′ Enlarged views of the pink boxes in **a**′. **c**′ A large central staining block with faint radial arms (green arrows). **d**′ A myelin-associated spheroid with clear profile (black arrow) and a blurred spheroid lacking an associated myelinated fiber (white triangle arrow). Scale bar: 20 µm

We confirmed that fluorescent staining in step 1 did not alter brightfield myelin signals acquired in step 2 in either wild-type tissue (Fig. 3a–f) or 5xFAD tissue (Fig. 3m–q).

Under brightfield microscopy, myelRed stained myelinated fibers formed different distribution patterns, from large white matter to moderate and small bundles and individual fibers with varying diameters and trajectories (Fig. 3d, g-l). Fine structure details of nodes of Ranvier (Fig. 3d, j) and parallel and tangential bundles were also distinguishable, particularly at the corpus callosum border (Fig. 3k, l).

On 5xFAD mice brain sections, dsCMA co-registered AmG+Aβ deposits and myelin signals at single fiber level alignment, revealing a variety of spatial relationship between Aβ deposits and associated myelin defects (Fig. 3r, s, t). High-resolution analysis showed that some Aβ deposits were coexisting with myelinated fibers, while others had no myelin colocalization (Fig. 3u, v, w). Unusually, some Aβ deposits with central staining blocks created a distinct bull-eye-like pattern (Fig. 3v). When applied on human brain sections, dsCMA method also obtained similar outcomes (see later sections).

In short, the dsCMA method offered a robust approach for detailed investigation of myelin fiber morphology under both normal and pathological conditions, enhancing the capability to systematically study whole mouse brains or large human tissue sections at various pathological stages.

### Myelin morphology changes in the 5xFAD AD model at different ages

To examine myelin alterations associated with AD pathogenesis, we analyzed age-dependent changes in the spatial relationships between myelinated fibers and Aβ deposits in the 5xFAD model at 2–3, 7+, and 12+ months of age, focusing on the cortex (Fig. 4) and hippocampus (Fig. 5).

In the cortex, at 2 months, sparse Aβ deposits were evident with well-defined cores but less halos (Fig. 4a, d). Gross myelin integrity appeared unaffected (Fig. 4b, c). Under high resolution, myelinated fibers maintained their morphologies even close to Aβ cores (Fig. 4e, f). No vascular Aβ presence was detected (Figs. 4a, b, c).

By 7 months, a noticeable increase in Aβ deposits was observed across the cortex, displaying diverse sizes and staining characteristics (Fig. 4g-j). Aβ-positive, blood vessel-like structures were also identified (Fig. 4g-i, orange arrows). Myelinated fibers’ distribution and regular trajectories were disrupted at the locations of Aβ deposits (Fig. 4i). Under high resolution, some Aβ deposits location showed a blank background with central staining block, a unique bullseye-like morphology, with myelin fibers either deflected peripherally (Figs. 4k, l; orange arrows) or unaffected (Figs. 4k, l; green arrow). At 12 months, these myelin disturbances became more pronounced, reflecting advanced pathology (data not shown).

In the hippocampus, at 2 months, Aβ deposits were more abundant compared to the cortex, with no substantial impact on myelin distribution pattern and trajectories (Fig. 5a–c). High-resolution imaging showed no deflection or disruption of myelin trajectories, various Aβ deposits types colocalized with morphologically normal myelinated fibers (Fig. 5d–f). Notably, no Aβ deposits were detected in the white matter at this stage.

By 7 months, the hippocampus exhibited a marked increase in Aβ number and diversity, particularly affecting subicular regions (Figs. 5g–i). The white matter was also affected at this stage, Aβ deposits in white matter were found associated with local myelin defects (Fig. 5j–l; orange arrows). High-resolution imaging revealed more myelinated fiber abnormality associated with Aβ deposits, including large spheroids and varicosities on fibers (Fig. 5m–p and q–t, arrows). In white matter, Aβ deposits coincided with myelin defects which were characterized by bullseye-like staining blocks, myelin-linked or independent staining blocks were also found (Fig. 5q–t). The spatial relationship of the staining blocks and myelinated fibers implicated residual debris from degeneration or broken myelin fragments (Fig. 5u–y and z–d’).

At 12 months, the distribution of neurite spheroids became denser in the hippocampus compared to the cortex, correlating with more pronounced myelin damage, particularly in ventral regions (Extended Fig. 5a–c and l–n; Supplementary Information). These neurite spheroids varied in size and morphology, interspersed with intact myelinated fibers near Aβ deposits, and exhibited diverse staining intensities and patterns (Extended Fig. 5d–g and h–k). High-resolution imaging revealed that various changes were found in the ventral cortex and hippocampus (Extended Fig. 5o–r, s–v, and w–z), underscoring the progressive, region-specific nature of AD pathogenesis.

Overall, dsCMA pipeline revealed that Aβ deposits number and diversity increased in cortex and hippocampus from young to middle age in 5xFAD mice, coinciding with increased abnormality and defects of myelin fibers. These abnormalities and defects were temporal and regional depending, with mild defects occurring at 2 months. The damage occurred earlier in the hippocampus, and progressed rapidly and more severely compared to the cortex, including white matter damage. These findings underscore the critical importance of spatial and temporal dynamics in AD pathology.

### Quantitative analysis of time course correlations between amyloid plaques and myelinated axons in 5xFAD mice

**Fig. 6:**
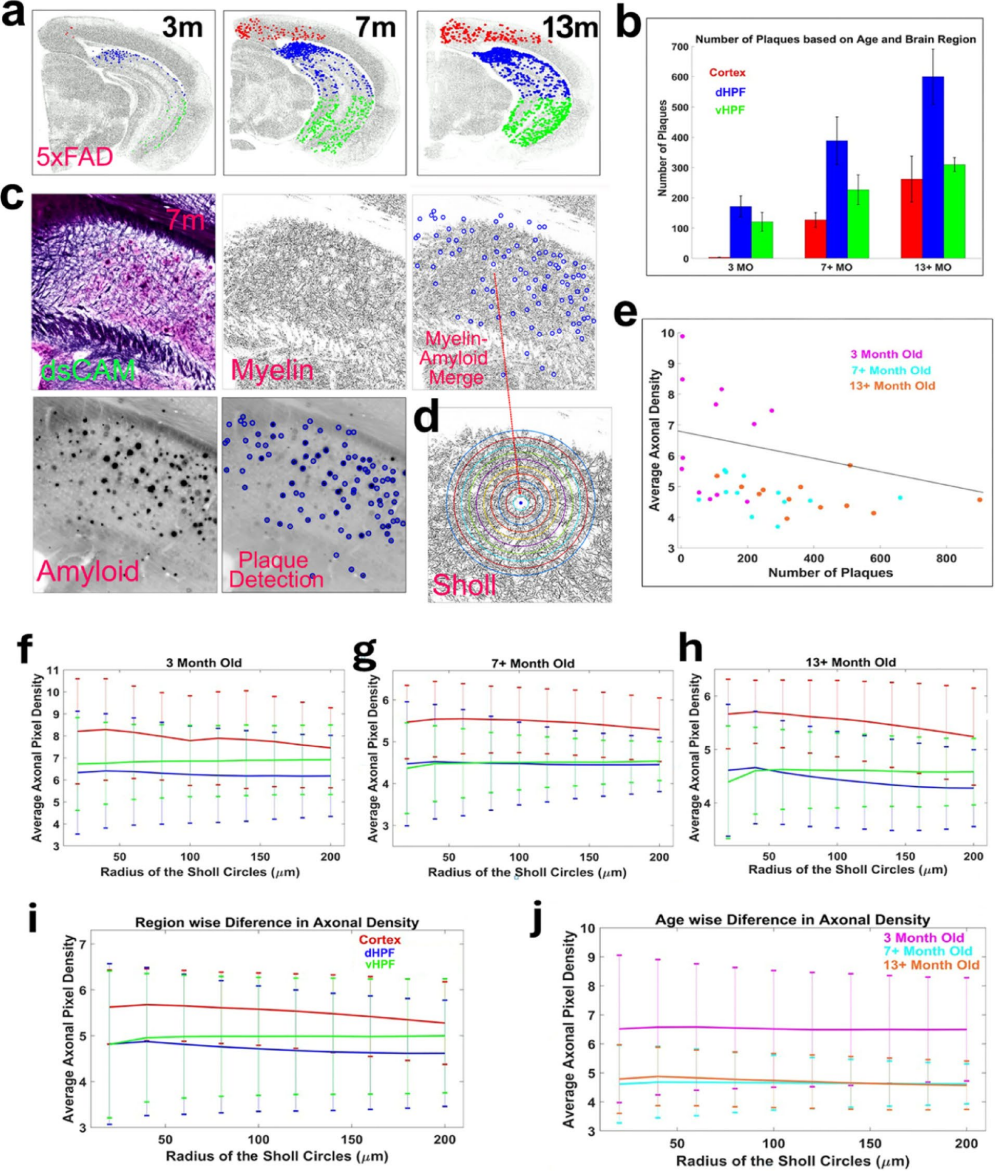
Quantification of amyloid plaques and surrounding myelinated axons in 5xFAD mice. **a** Representative images showing identified plaques (Aβ signal) overlaid on myelinated single-axon signals in 3-month-old (left, 3 males and 1 female), 7-month-old (middle, 2 males and 2 females), and 13-month-old (right, 2 males and 2 femals) 5xFAD mice (*n* = 4 per group). **b** Bar plot showing progressive increases in plaque number from 3 to 7 to 13 months across cortex (red), dorsal hippocampal formation (dHPF; blue), and ventral hippocampal formation (vHPF; green). **c** Workflow for quantifying axonal density surrounding plaque locations. dsCMA images show myelinated axons (dark purple/black) intermingled with plaques (upper left), and corresponding AmG Aβ labeling identifies plaques as dark spots on a white background (lower left). Myelinated axons in gray matter were thresholded using a Frangi filter (upper middle). Plaques were detected using a semi-automated approach (Laplacian-of-Gaussian filtering followed by manual editing; lower middle). Plaque locations were projected onto the axon image to generate composite images (upper right) used for Sholl analysis (**d**). Concentric rings were generated at 20-µm intervals (20–200 µm radius), and axonal density was calculated as myelinated axonal pixels per ring area. Red arrow indicates the same plaque location in **b** and **c**. **e** Scatter plot showing regional average axonal density versus total plaque number, color-coded by age (3 months, magenta; 7 months, cyan blue; 13 months, orange). **f**–**h** Sholl-based axonal density profiles in cortex (red), dHPF (blue), and vHPF (green) in 3-month-old (**f**), 7-month-old (**g**), and 13-month-old (**h**) mice. **i** Overall axonal density comparison across regions pooled across ages (cortex, red; dHPF, blue; vHPF, green). **j** Overall axonal density comparison across age groups pooled across regions (3 months, magenta; 7 months, cyan blue; 13 months, orange).

We further conducted a quantitative analysis of the spatial correlations between amyloid plaques and myelinated axons in 5xFAD mice across three age groups: 3 months (3 m, *n* = 4, 3 males and 1 female), 7 months (7 m, *n* = 4, 2 males and 2 females), and 13 months (13 m, *n* = 4, 2 males and 2 females) (Fig. 6). Specifically, we focused on three regions at the same coronal level: the cortex (CTX, primarily the retrosplenial area or RSP), the dorsal hippocampus (dHPF), and the ventral hippocampus (vHPF) (see Methods section). Our analysis was performed at two levels. First, at the individual sample/animal level, we quantified the number of amyloid plaques in each age group and region (Fig. 6a, b). Second, we conducted a comparative analysis at the individual plaque level to assess spatial relationships between plaques and myelinated axons (Fig. 6c–j).

*Plaque count comparison across regions and age groups.* We first compared the number of amyloid plaques in the three regions of interest in 3 m 5xFAD mice (Fig. 6a, b). A significant difference in plaque number was observed among these regions, Cortex (primarily RSP), dHPF, and vHPF (Kruskal–Wallis test, *p* < 0.05). The cortex exhibited a very low plaque count (average 4 ± 1) compared to a significantly higher number in the dHPF (average 172 ± 70, *p* < 0.05, Wilcoxon rank-sum test) and vHPF (average 121 ± 62, *p* < 0.05, Wilcoxon rank-sum test). However, no substantial difference was found between dHPF and vHPF in the 3 m animals (*p* > 0.05).

In 7 m mice (Fig. 6a, b), we observed a significant increase in plaque count in both the cortex (average 127 ± 48, *p* < 0.05) and dHPF (average 389 ± 157, *p* < 0.05) compared to the corresponding regions in 3 m mice. However, vHPF (average 227 ± 98) did not show a substantial increase. Within the 7 m group, the dHPF exhibited a significantly higher number of plaques than the cortex, while no substantial differences were observed between the cortex and vHPF or between vHPF and dHPF.

While there were trends of increased plaque quantity, the comparisons between the 7 m and 13 m groups at the level of individual regions did not reveal substantial increase in number of plaques (Wilcoxon rank-sum test, *p* > 0.05). Within the 13 m group (Fig. 6a,b), the dHPF (average 600 ± 211) exhibited a significantly higher plaque count than the vHPF (average 310 ± 53, *p* < 0.05), but neither region displayed a substantially higher plaque count than the cortex (average 261 ± 175).

We next analyzed the total plaque count across all age groups within each region (Fig. 6b), revealing a significant difference between regions (Kruskal–Wallis test, *p* < 0.05). Pairwise comparisons showed that each region had a significantly different plaque count from the other two (Wilcoxon rank-sum test, *p* < 0.05). Finally, we examined the overall changes in plaque numbers across age groups, summing across all three regions. The 3 m group had a significantly lower total plaque count compared to both the 7 m and 13 m groups (Wilcoxon rank-sum test, *p* < 0.05), while no substantial difference was observed between the 7 m and 13 m groups (Fig. 6b).

*Analysis of myelinated axonal fiber distribution surrounding individual plaques.* We quantified the impact of amyloid plaques on surrounding myelinated axons (Fig. 6c–j). First, we assessed the relative influence of plaque abundance on regional connectivity (Fig. 6e). A significant negative correlation was observed between the total number of plaques in a region and the average axonal density surrounding each plaque (Pearson’s correlation, *R* = -0.42, *p* < 0.05). Additionally, axonal density declined significantly with increasing age (*R* = -0.51, *p* < 0.05).

Next, we examined each age group separately to quantify the impact of individual plaques on myelinated axons (Fig. 6c, d). Using a Sholl-like analysis, we measured myelinated axonal density surrounding each plaque by calculating the number of pixels per unit area within each Sholl circular band, with radii increasing in 20 µm intervals. The number of pixels within each band was normalized by dividing it by the area of the band. The overall surrounding axonal density was determined by averaging axonal densities across all Sholl bands (ranging from 20 to 200 µm).

In 3 m 5xFAD mice, axonal density varied substantially across the three regions (Fig. 6f). The cortex exhibited the highest axonal density, followed by the vHPF, with the dHPF showing the lowest density (significant differences in all pairwise comparisons, Student’s *t*-test, *p* < 0.05). By 7 m, axonal connectivity had declined significantly in all three regions compared to their counterparts in 3 m animals (*p* < 0.05). Within the 7 m group, the cortex retained higher axonal density than both the vHPF and dHPF (*p* < 0.05), but no substantial difference was observed between the vHPF and dHPF (Fig. 6g).

Comparisons between 7- and 13-month-old mice revealed no substantial change in axonal density in the cortex, whereas the dHPF showed a modest decreasing trend. In contrast, axonal density in the vHPF exhibited a slight increase from 7 to 13 months. Notably, in 13-month-old mice, all three regions displayed significantly different axonal densities (Fig. 6h), with the cortex showing the highest density, followed by the vHPF, and the dHPF the lowest—mirroring the regional pattern observed at 3 months.

When all age groups were combined, the cortex consistently exhibited the highest axonal density, followed by the vHPF, whereas the dHPF displayed the lowest density (Fig. 6i). Overall axonal density declined markedly from 3 months, with 3-month-old mice showing substantially higher axonal density than both 7- and 13-month-old mice, which exhibited comparable levels (Fig. 6j).

### Aβ deposits and myelin changes in AD human brain tissue

We further applied the dsCMA pipeline to human AD brain tissue, analyzing samples from two patients: one 29-year-old male with hereditary early-onset AD and cerebrovascular disease, and one 91-year-old male with sporadic AD. Our goal was to investigate whether human AD patients exhibit similar myelin pathologies to those observed in the 5xFAD mouse model. If such parallels exist, dsCMA could serve as an effective method for systematically exploring the neuropathological landscape of human AD, thereby advancing our understanding of its mechanisms. We focused on the cortex and hippocampus in human tissues to mirror the regions studied in the 5xFAD mouse model.

#### Gray and white matter showed different myelin defect patterns in AD brain tissue

On the first early-onset AD patient temporal lobe sample, our data revealed myelin deterioration within both the white and gray matter in the early disease stage.

**Fig. 7:**
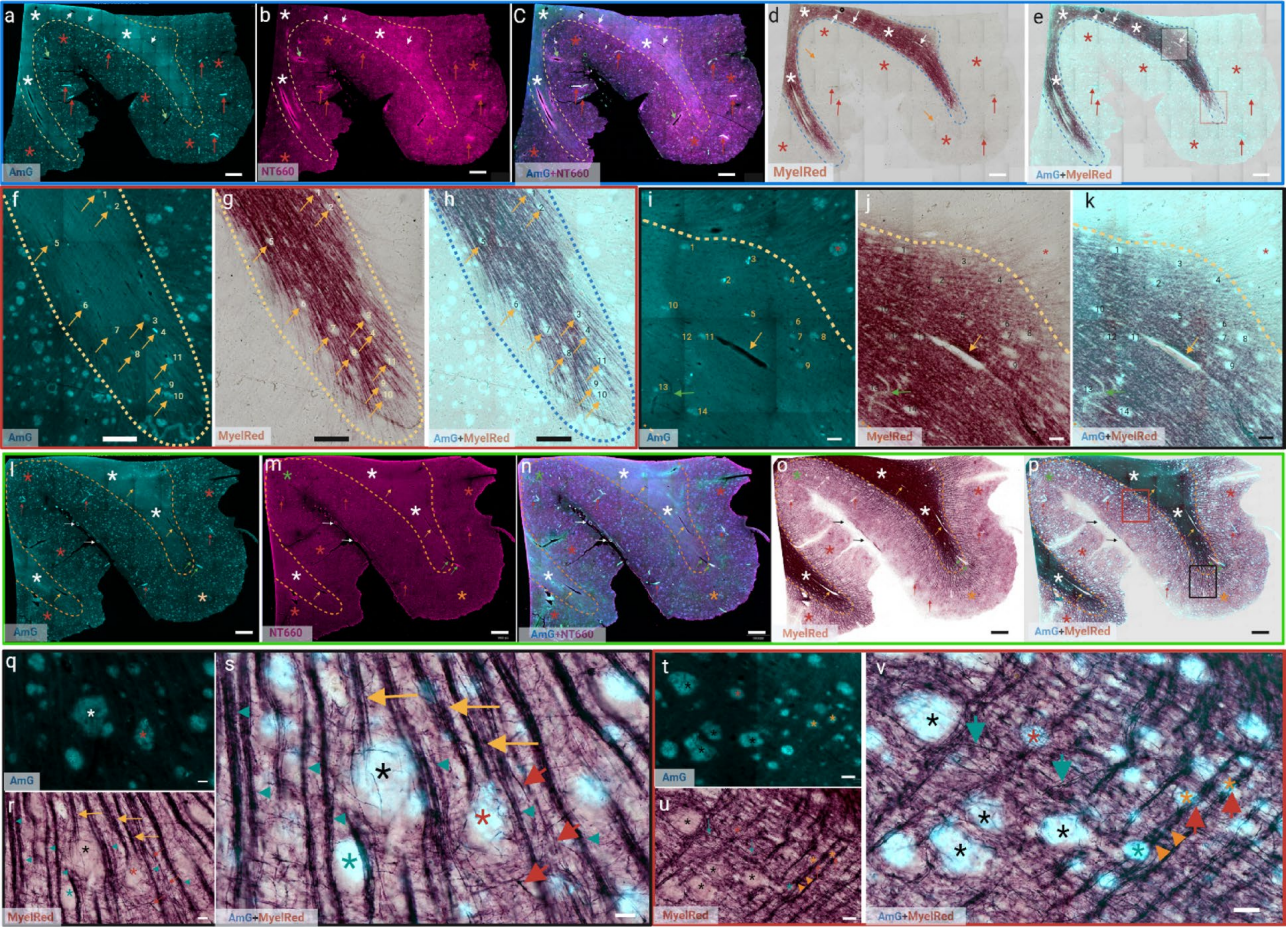
Spatial relationship between myelinated fibers and Aβ deposits in a familial AD patient brain (29 years old). **a**–**k** (white matter damage). Dashed yellow line indicates the gray–white matter boundary. **a** Thin Sect. (25 µm) showing abundant AmG+Aβ deposits in gray matter (red stars) and sparse deposits in white matter (white arrows; white star). AmG+ (red arrows) and AmG − (green arrows) vessel-like structures indicate cerebral amyloid angiopathy (CAA)-like pathology. **b** Nissl staining of the same section showing corresponding vessel-like structures (arrows). **c** Co-registration of **a** and **b**. **d** Brightfield MyelRed staining of the same section. **e** Warp-registered overlay of **a** and **d** showing spatial relationships among Aβ deposits, vessel-like structures, and myelinated fibers. Scale bar: 750 µm. **f**–**h** Enlarged view of red box in **e**. **f** Aβ deposits in white matter and gyral radial fibers (orange arrows and numerals), aligned with white matter bundle trajectories. **g** Corresponding myelin image showing localized myelin defects at Aβ deposit sites (orange arrows and numerals). **h** Co-registration of **f** and **g** highlighting spatial correspondence between deposits and myelin defects. Scale bar: 250 µm. **i**–**k** Enlarged view of black box in **e** (gyri–sulci transition). **i** Dense-core and diffuse Aβ deposits aligned with fiber trajectories (numerals), including a large round deposit among loosely organized radial fibers in gray matter (red star). Branched (green arrow) and slit-like (orange arrow) vessel-like structures are indicated. **j** Corresponding myelin image showing localized myelin defects at Aβ deposit sites (numerals) in both white and gray matter (red star). **k** Co-registration showing one-to-one alignment between Aβ deposits and myelin defects. Scale bar: 100 µm. **l**–**v** (gray matter damage). Dashed yellow line indicates the gray–white matter boundary. **l** Thick Sect. (40 µm) showing CAA-like structures on the brain surface (white arrows) and within gray matter (red arrows). A vessel-like structure traversing gray and white matter shows CAA-like staining only in the gray matter segment (green arrows) but not in white matter (orange arrows). **m** Nissl staining of the same region. **n** Co-registration of **l** and **m**. **o** Myelinated fiber morphology in the same region shows a gradual transition from long gyral radial fibers (orange star) to short sulcal parallel fibers (green star), with reduced fiber length from gyri to sulci. **p** Co-registration of **l** and **o** showing spatial associations between Aβ/CAA-like pathology and myelinated fibers (symbols correspond to those in **l** and **o**). Scale bar: 750 µm. **q**–**s** Enlarged view of black box in **p** (gyral region). **q** Aβ deposits with variable size and morphology (stars). **r** Corresponding myelin image showing blank spots at deposit locations, with radial bundle trajectories either disrupted (orange arrows and stars) or preserved/mildly altered (green triangular arrows). Normal-appearing individual myelinated fibers are present at and near deposits (black/red stars; red arrows). **s** Co-registration showing Aβ deposits with long-axis orientation aligned with gyral radial fiber bundles. Scale bar: 25 µm. **t**–**v** Enlarged view of red box in **p** (sulcal region). **t** Aβ deposits of varied size and morphology (stars). **u** Corresponding myelin image showing parallel bundle trajectories either disrupted (green and orange triangles) or preserved (orange stars; red arrows), with normal-appearing individual fibers present at deposit sites (blue arrows; black/green stars). **v** Co-registration showing deposits with long axes oriented perpendicular to sulcal parallel fiber bundles. Scale bar: 50 µm

For white matter, 25 µm thick sections were used to avoid signal saturation and enabled detailed visualization of myelin patterns along the white–gray matter continuum (Fig. 7a–k). The white matter deposits were smaller, faintly stained, and predominantly oval shaped, aligning with myelin trajectories (Fig. 7a, f, i). These deposits progressively expanded their size from densely myelinated areas towards sparser regions, from deep white matter to gyri radial fibers and coincided with local myelinated fibers defects (Fig. 7f–k). No evidence of cerebral amyloid angiopathy in white matter was observed.

For gray matter, we analyzed 50 µm sections to capture the heterogeneous distribution of Aβ deposits (Fig. 7l–v). The gray matter Aβ deposits showed diverse size and morphologies, uniformly scattered, forming a cobblestone-like pattern within the cortical matrix (Fig. 7l). Fibers and bundles extending from the white matter gradually shortened, displaying a tapered continuity that transitioned from the gyri summits to the sulci depths (Fig. 7o). Additionally, cerebral amyloid angiopathy (CAA)-like structures were identified within the gray matter and along the pial surface. A single blood vessel-like structure penetrated both gray and white matter; however, AmG signals were exclusively observed in the gray matter portion, with no pathological manifestations in the white matter (Fig. 7l–p, green arrows).

The spatial relationship between Aβ deposits and myelinated fibers in both white and gray matter revealed abnormalities in bundle density and integrity (Fig. 7l, o, p). High-resolution imaging in the gyri summit region showed that intact myelinated fibers exhibited slight contouring away from Aβ deposits, with the deposits’ long axis aligning with radial trajectories of the gyri (Fig. 7q, r, s). In contrast, in the sulci region, the long axis of Aβ deposits was oriented vertically relative to the parallel bundles (Fig. 7t, u, v). This intricate spatial interplay between Aβ deposits and myelinated fibers existed across different regions, resembling those observed in early stage 5xFAD mice (Figs. 7s, v).

**Fig. 8:**
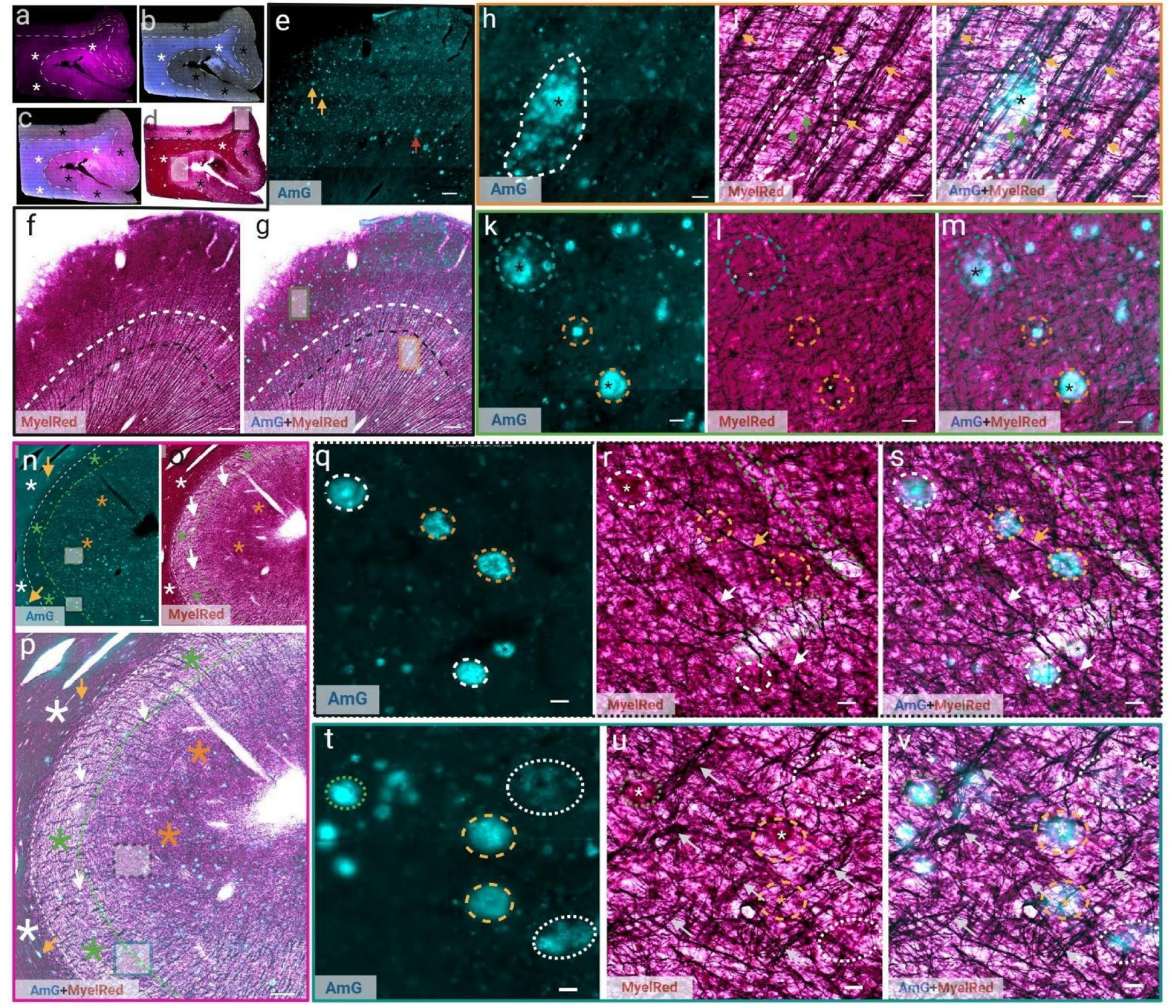
Spatial relationship between Aβ deposits and myelinated fibers in the cortex of a sporadic AD patient (91 years old). **a**–**d** Application of dsCMA to the same cortical section. **a** Nissl staining highlights cytoarchitecture. **b** Amylo-Glo (AmG) staining identifies Aβ deposits. **c** Co-registration of **a** and **b**. **d** Brightfield MyelRed staining reveals myeloarchitecture. The gray–white matter boundary is indicated (white dashed line), with white matter (white star) and gray matter (black star) marked. The inner (orange dashed line) and outer (green dashed line) bands of Baillarger are indicated. **e**–**g** Enlarged view of the black box in **d** showing gyral radial myelinated fiber bundles and associated Aβ deposits. Outer (white dashed line) and inner (black dashed line) boundaries of the band of Baillarger are indicated. **e** Aβ deposits of varying size and morphology distributed near the cortical surface (orange arrows) and in deeper layers (red arrow). **f** Corresponding myelin image showing radial myelinated bundles across cortical layers. **g** Co-registration of **e** and **f**. Orange and green boxes highlight Aβ deposits shown in **e**. Scale bar: 250 µm. **h**–**j** Enlarged view of the orange box in **g**. **h** Numerous Aβ deposits with variable size, morphology, and intensity, including a large dense-core plaque associated with multiple eccentric smaller cores (white dashed outline). **i** Corresponding myelin image showing parallel bundles (orange arrows) and normal-appearing individual fibers (green arrows) at the plaque location. **j** Co-registration of **h** and **i**, showing no substantial disruption of individual fibers or bundles within the Aβ deposit. **k**–**m** Enlarged view of the green box in **g**. **k** Aβ deposits with diverse morphologies, including dense cores (orange dashed circles) and complex irregular plaques containing multiple dense cores (green dashed circle). Dense-core substructures are indicated (black stars). **l** Corresponding myelin image. **m** Co-registration of **k** and **l** showing preserved fiber distribution and orientation at plaque sites (green/orange dashed outlines), with small staining blocks indicated (white stars). Scale bar: 25 µm. **n**–**p** Enlarged view of the pink box in **d**. White dashed line indicates the gray–white matter boundary. **n** Aβ deposits are sparse in white matter (white star) and abundant in gray matter (orange star), with deposit long axes aligned with myelin bundle trajectories (orange arrows). A deep sulcal transition layer (green stars; between dashed white and green lines) shows reduced Aβ deposition compared to superficial gray matter (orange stars). **o** Corresponding myelin image showing the transition layer enriched with short parallel bundles (white arrows) intermingled with abundant U-shaped fibers (green stars), whereas the superficial layer contains smaller bundles and individual fibers without a dominant orientation. **p** Co-registration of **n** and **o** showing white matter Aβ deposits associated with local myelin defects (orange arrows). Scale bar: 250 µm. **q**–**s** Enlarged view of the black dashed box in **p**. **q** Coexisting plaque types, including small dense-core plaques with large halos (upper white dashed circles), large dense-core plaques without halos (lower white dashed circle), plaques with dense-core substructures (orange dashed circles), and small dense-core plaques without halos (black star). **r** Corresponding myelin image showing normal-appearing fiber bundles colocalized with vessel-like structures (green dashed lines; white arrow) and traversing Aβ deposits (orange/white dashed circles and arrows) without detectable abnormalities. **s** Co-registration of **q** and **r**. Scale bar: 25 µm. **t**–**v** Enlarged view of the blue box in **p**. **t** Coexisting plaque types, including diffuse deposits (white dashed circles), small dense-core plaques with large halos (orange dashed circles), and large dense-core plaques with small halos (green dashed circle). **u** Corresponding myelin image. **v** Co-registration of **t** and **u** showing heterogeneous plaque-associated myelin changes, including staining blocks (white star) and locally reduced fiber density at some deposits, whereas other deposits show no detectable myelin abnormalities (yellow star; orange/white dashed circles). Parallel sulcal bundles (white arrows) and individual fibers show no substantial orientation changes. Scale bar: 25 µm

On the second late-onset sporadic AD patient tissue, distinct patterns of Aβ-myelin interaction were found across cortical subregions (Fig. 8a–g). In the deep layer of the gyral crest, large Aβ deposits with complex inner structures co-localized with parallel myelin bundles and individual fibers, retained their morphology, orientation, and trajectories, with no noticeable morphology defects (Fig. 8h–j; green and orange arrows), and density reduction (Fig. 8g, h–j; black star and white dashed circle). In the top layer of the gyral crest, Aβ deposits of varying sizes and morphologies were identified, again their associated or adjacent myelinated fibers showed no detectable alternations, abnormalities or defects (Fig. 8k–m; circles).

Conversely, in the cortical sulci, some Aβ deposits were associated with reduced myelin fiber density in the adjacent regions (Fig. 8n–p and q–v; white star). Despite this, the majority of Aβ deposits in these regions were surrounded by normal-appearing myelinated fibers and bundles, with no observable alterations in their morphology or trajectories (Fig. 8q–s and t–v; circles and yellow star).

In summary, using the dsCMA approach, we found that the minimal myelinated fiber defects observed in the second sporadic AD patient’s cortex contrasted sharply with the pronounced abnormalities seen in the first early-onset AD case. This inter-patient and inter-regional comparison underscores the heterogeneous nature of Alzheimer’s disease pathology and the complex interplay between Aβ deposition and myelin integrity within the human AD cortex.

#### Hippocampal Aβ deposits and myelin morphology displayed distinct spatial relationships compared to the cortex in the same patient

**Fig. 9:**
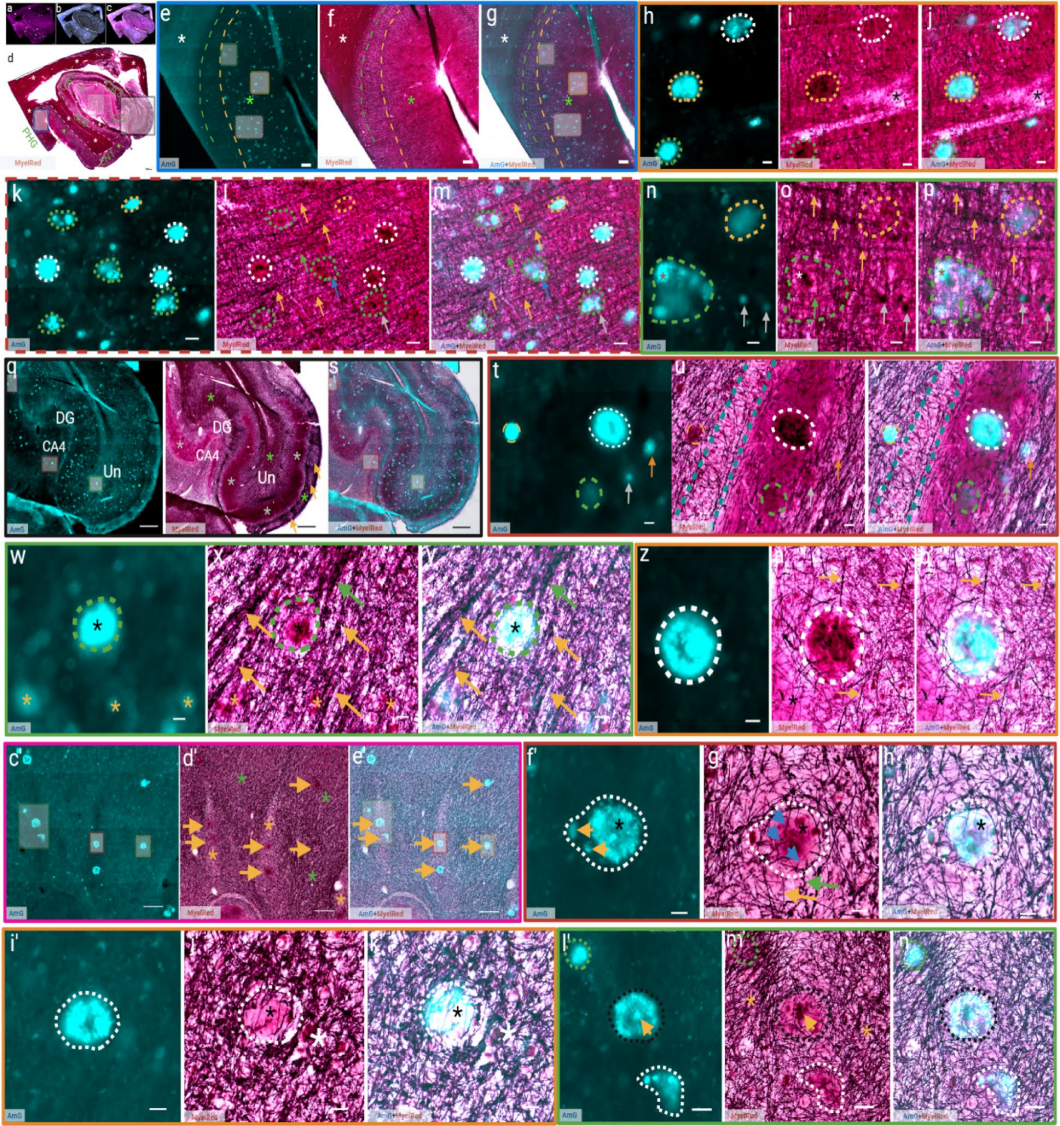
Spatial relationship between Aβ deposits and myelinated fibers in the hippocampus of a sporadic AD patient (91 years old). **a**–**d** dsCMA applied to the same hippocampal section. **a** Nissl staining highlights cytoarchitecture. **b** Amylo-Glo (AmG) staining identifies Aβ deposits. **c** Co-registration of **a** and **b**. **d** Brightfield MyelRed staining reveals myeloarchitecture. Gray–white matter boundary is indicated (white dashed line), with white matter (white stars) and gray matter (green stars) marked. Inner (green dashed line) and outer boundaries of the band of Baillarger are indicated. Abbreviations: PHG, parahippocampal gyrus; ENT, entorhinal cortex; SUB, subiculum; DG, dentate gyrus; cs, collateral sulcus. Scale bar: 2000 µm. **e**–**g** Enlarged view of the blue box in **d**. **e** Numerous small, low-intensity Aβ deposits are present in a deep-layer transitional zone of PHG (between dashed orange and green lines), whereas larger and morphologically diverse deposits are enriched in the superficial layer (green star). **f** Corresponding myelin image. **g** Co-registration of **e** and **f** shows decreasing density of individual fibers and small radial/parallel bundles from deep to superficial layers. Aβ deposits are sparse in white matter (white star) but abundant in gray matter (green star). Scale bar: 250 µm. **h**–**j** Enlarged view of the orange box in **g** (superficial gyral layer). **h** Coexisting Aβ deposit types include large dense-core plaques with small halos (yellow dashed circle), diffuse coarse granular deposits (white dashed circle), large dense-core plaques without halos (green dashed circle), and small dense deposits (white star). **i** Corresponding myelin image. **j** Co-registration shows disrupted fiber trajectories and residual dark staining blocks at some deposit sites (yellow and green dashed circles), whereas other deposits show reduced fiber density without obvious disruption or debris (white star; white dashed circle). Vessel-like structures are indicated (black star). Scale bar: 50 µm. **k**–**m** Enlarged view of the red dashed box in **g** (deep PHG layer). **k** Multiple Aβ morphologies coexist, including large and small dense-core plaques without halos (white dashed circles; yellow dashed circle) and plaques with variable halo size (green dashed circles). **l** Corresponding myelin image. **m** Co-registration shows heterogeneous plaque-associated myelin changes: local reduction of individual fibers and small bundles (yellow arrows) with minimal or sparse debris (green dashed circles; gray arrow), marked disruption of radial bundles with debris clusters (white dashed circles), as well as mild (yellow dashed circle) or absent abnormalities (blue arrow; lower-left green dashed circle). Scale bar: 50 µm. **n**–**p** Enlarged view of the green box in **g** (deep sulcal region). **n** Aβ deposits include a large complex plaque with multiple dense cores (red star), a large halo region between cores (green dashed circle), a diffuse deposit lacking dense core (yellow dashed circle), and multiple tiny deposits (gray arrows). **o** Corresponding myelin image. **p** Co-registration shows that the large complex plaque is associated with disrupted fibers (green dashed circle), debris staining blocks (white star), and adjacent normal fibers (green arrow). Diffuse deposits show reduced fiber density without disruption or debris (yellow dashed circle). Tiny deposits show debris staining blocks (gray arrows), with no substantial change in parallel bundles (yellow arrows). Scale bar: 50 µm. **q**–**s** Enlarged view of the black box in **d** (DG and uncus, Un). **q** Aβ deposits of variable size, morphology, and intensity extend from superficial to deep layers. **r** Corresponding myelin image. **s** Co-registration shows Aβ deposits distributed across myelin-dense (green stars) and myelin-sparse (white stars) zones, including alternating dense and sparse myelin regions along the Un surface (yellow arrows). Scale bar: 750 µm. **t**–**v** Enlarged view of the red box in **s**. **t** Aβ deposits include small dense-core plaques without halos (yellow dashed circles), large dense-core plaques with small halos and internal slits (white dashed circle), diffuse deposits lacking dense core (green dashed circle), tiny dense-core plaques with large halos (gray arrow), and large dense-core plaques with small halos (yellow arrow). **u** Corresponding myelin image. **v** Co-registration shows diverse plaque-associated myelin phenotypes, including preserved fibers with high background staining (yellow dashed circle), debris staining blocks without detectable fibers (white dashed circle), sparse fibers without debris (green dashed circle), preserved fibers (yellow arrow), or moderate fiber reduction with blurred profiles (gray arrow). Blue dashed lines indicate the DG cell layer. Scale bar: 25 µm. **w**–**y** Enlarged view of the green box in **s**. **w** Aβ deposits include a large dense-core plaque with thin halo (black star; green dashed circle) and diffuse deposits with blurred halos (yellow stars). **x** Corresponding myelin image. **y** Co-registration shows abundant tortuous and varicose fibers and parallel bundles (yellow arrows) forming an entangled network. Plaque sites show disrupted trajectories (green arrow; green dashed circle) or reduced density with increased red background (black and white stars), without prominent debris blocks. Scale bar: 25 µm. **z**–**b**′ Enlarged view of the orange box in **s**. **z** Large dense-core thin-halo plaque with internal slits (white dashed circle). **a**′ Corresponding myelin image. **b**′ Co-registration shows disrupted fiber trajectories with sparse residual fibers at the plaque edge and clustered debris staining blocks (white dashed circle). Varicose fibers of variable thickness are indicated (yellow arrows; black star). Scale bar: 25 µm. **c**′–**e**′ Enlarged view of the purple box in **d**. **c**′ Numerous small deposits coexist with several large dense-core plaques containing internal slits. **d**′ Corresponding myelin image. **e**′ Co-registration shows myelin-sparse regions (yellow stars) interspersed with dense regions (green stars), with reduced fiber density and increased red background at plaque sites (yellow arrows). Scale bar: 250 µm. **f**′–**h**′ Enlarged view of the red box in **e**′. **f**′ Large complex Aβ deposit (white dashed circle) composed of multiple granular dense cores without halo (black star) and accompanied by smaller deposits (yellow arrows). **g**′ Corresponding myelin image. **h**′ Co-registration shows reduced fiber density at the plaque site with sparse residual fibers (blue arrows) and faint debris staining blocks (black star), with coexisting normal (yellow arrow) and varicose fibers (green arrow). Scale bar: 25 µm. **i**′–**k**′ Enlarged view of the orange box in **e**′. **i**′ Large dense-core thin-halo plaque with internal slits (white dashed circle). **j**′ Corresponding myelin image. **k**′ Co-registration shows disrupted fiber trajectories with few residual fibers and no prominent debris staining blocks (black star), while adjacent regions contain entangled varicose fibers obscuring individual morphologies (white star). Scale bar: 25 µm. **l**′–**n**′ Enlarged view of the green box in **e**′. **l**′ Aβ deposits include a small plaque with double dense cores and thin halo (green dashed circle), a large plaque with central halo and multiple peripheral cores (black dashed circle; yellow arrow), and a plaque with three dense cores and diffuse halo (white dashed circle). **m**′ Corresponding myelin image. **n**′ Co-registration shows preserved fiber density and trajectories at plaques within myelin-dense regions (yellow stars; green dashed circle). In contrast, plaques in myelin-sparse regions show increased red background, mild density reduction (white and black dashed circles), and central debris staining blocks (yellow arrow). Scale bar: 50 µm

The hippocampus has been recognized as an early target in AD progression, we investigated the Aβ deposits and myelin spatial interactions across hippocampal subregions in the second patient (Fig. 9a–d). We found diverse morphologies and densities of Aβ deposits colocalized with varying degrees of myelin abnormalities (Fig. 9e–g).

In the parahippocampal gyrus (PHG) region, the superficial layers contained Aβ deposits with staining blocks surrounded by normal-appearing myelinated fibers, which exhibited random trajectories with no dominant orientation (Fig. 9h–j). The intermediate layers showed disrupted myelin bundles at dense-core Aβ deposits associated with staining blocks (Fig. 9k–m; white dashed circles). However, adjacent Aβ deposits were colocalized with normal-appearing myelinated fibers, displaying no staining blocks or visible defects (Fig. 9l, m; green dashed circles). In the deep layers, diffuse Aβ deposits were surrounded by intact myelinated fibers with no evident defects at the single fiber or bundle level (Fig. 9n–p; orange dashed circles and arrows). Some complex structure and small diffuse Aβ deposits were colocalized with disrupted myelin staining blocks (Fig. 9n–p; green dashed circles and gray arrows), while the same complex Aβ deposits were also associated with normal myelinated fibers nearby (Fig. 9n–p; green arrow).

Further investigation into the subiculum, CA1, and dentate gyrus (DG) regions revealed variations in Aβ-myelin interactions with distinct regional features (Fig. 9q–s). For instance, the DG region exhibited myelin defects coinciding with Aβ deposits of varying sizes (Fig. 9t–v), while the subiculum showed interrupted myelin bundle trajectories caused by Aβ deposits, but without substantial alterations to the surrounding myelin (Fig. 9w–y; green circle and black star).

In the CA1 region, sparse complex Aβ structures were observed amidst intact fibers and numerous staining blocks (Fig. 9z, a’, b’; white circle). Tortuous fibers and local swellings were prominent in these areas (Fig. 9z, a’, b’; orange arrows). In the CA4 region, Aβ deposits were distributed across both myelin-dense and myelin-sparse subregions (Fig. 9c’, d’, e’). Myelin fiber reduction was associated with Aβ deposits in both myelin-sparse (Fig. 9f’–h’ and l’–n’) and myelin-dense subregions (Fig. 9i’–k’), although residual normal-appearing fibers persisted.

The dense packing of tortuous fibers often complicated tracing individual trajectories (Fig. 9d’, j’). Notably, intact myelinated fibers were still colocalized with dense-core Aβ deposits in the myelin-dense subregion (Fig. 9l’–n’; green dashed circle).

We further analyzed pTau expression in the same patient to investigate neural degeneration (labeled by pTau) and associated myeloarchitecture changes. Increased pTau expression was concentrated along the long axis of the gray matter in the hippocampal region (Extended Data Fig. 6). However, each subregion displayed unique characteristics. In the parahippocampal gyri (PHG), the deep layers exhibited dense pTau-positive cells with degenerative profiles and neurites, but no substantial swelling was observed (Extended Data Fig. 6a). The top layers showed increased pTau patches that overlapped with Aβ deposits (41% Abeta colocalized with pTau) (Extended Data Fig. 6b,c,d). In the CA1 region, abundant pTau-positive degenerative neurons were observed without overlapping Aβ deposits (12.8% Abeta plaques colocalized with pTau) (Extended Data Fig. 6e,f,g). In the CA4 region, 24.7% Abeta with weak Aβ deposit profiles are colocalized with pTau clusters (Extended Data Fig. 6h–j). Notably, the pTau-degenerative areas did not exhibit substantial myelin degeneration compared to neighboring regions (data not shown).

In summary, our analyses of AD patient tissues revealed region-specific patterns of hippocampal myelin disruptions, which were not consistently associated with Aβ deposits. This suggests that other factors within the local microenvironment, such as neuron type, myelination status, inflammation, and the involvement of other cell types, may play critical roles in the observed pathologies. The pronounced differences in myelin alterations between the cortex and hippocampus within the same patient underscore the regional specificity of AD pathology. These distinctions at both the regional and fiber levels highlight the complexity of AD and emphasize the need for a nuanced investigation into its progression.

#### Heterogeneity in cortical myeloarchitecture revealed complex Aβ-myelin interaction

**Fig. 10:**
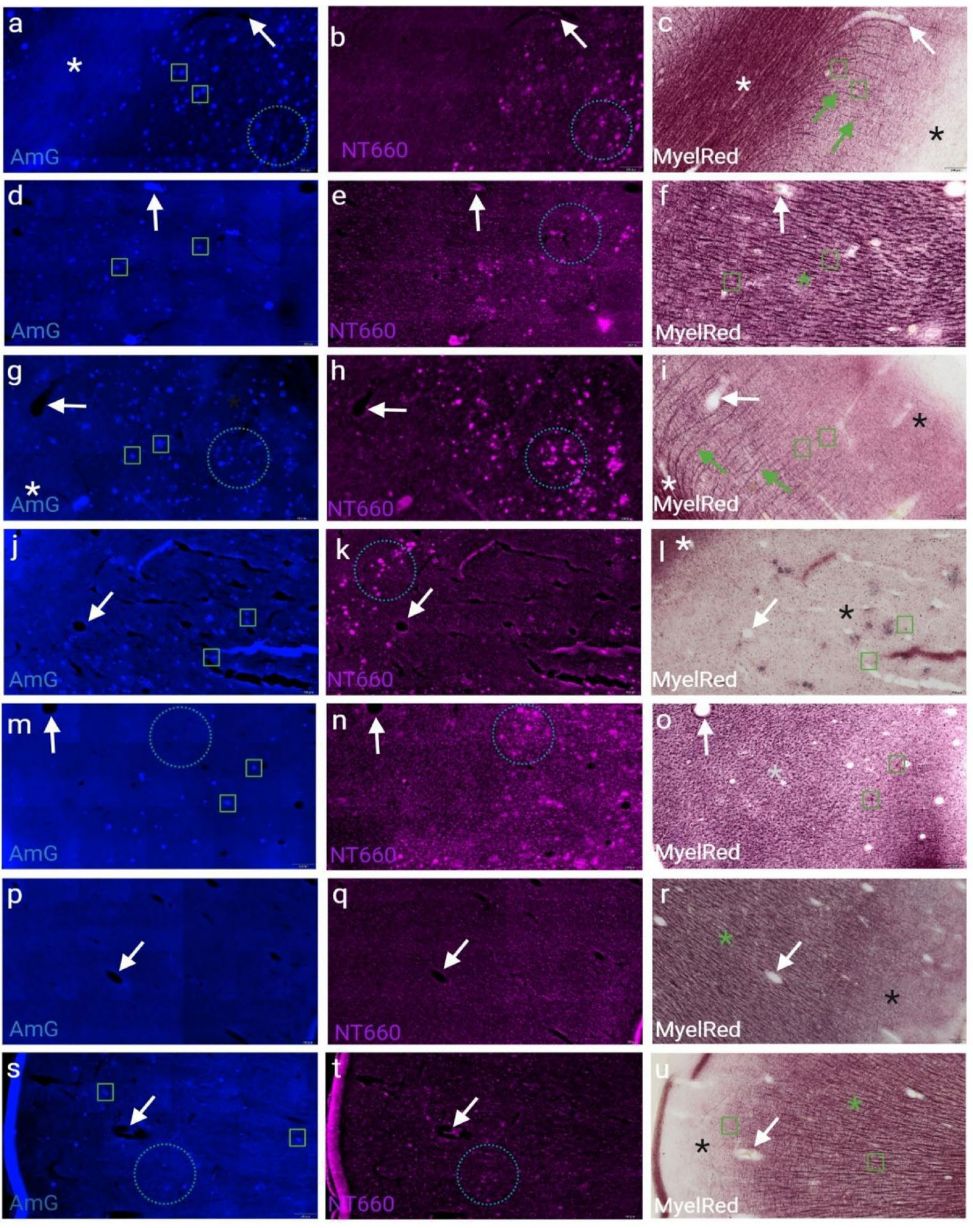
Heterogeneity of Aβ plaque pathology and myelin architecture in human cortical tissues. **a**–**u** Representative dsCMA-processed cortical sections from four AD patients (**a**–**l**), two diabetic non-AD patients (**m**–**o** and **s**–**u**), and one non-AD control (**p**–**r**). Columns show AmG-labeled Aβ plaques (left), NT660-labeled Nissl substance (middle), and MyelRed-labeled myelinated fibers (right). White arrows indicate blood vessels used as anatomical landmarks. Green squares mark regions of interest (ROIs) containing Aβ plaques. Green arrows indicate gray matter parallel fiber bundles, and green stars indicate white matter radial fibers within gyral cores. Black stars denote superficial gray matter layers, gray stars indicate clustered myelin bundles, and blue dashed circles highlight large Nissl-stained deposits. Across AD cases (**a**–**l**), Aβ pathology showed marked interindividual variability in plaque size (10–306.9 µm), cortical distribution (diffuse versus focal), and associated myelin organization (fiber density, orientation, and trajectory patterns). The non-AD control (**p**–**r**) exhibited minimal Aβ pathology with preserved myelin architecture. Diabetic non-AD cases (**m**–**o** and **s**–**u**) exhibited unexpected Aβ plaque deposition (arrowheads), with partial colocalization of NT660+ neuronal debris, suggesting potential metabolic contributions. Myelin heterogeneity was observed across all cases, including fragmented or faint “ghost fibers” in superficial layers (black stars), potentially reflecting age-related degeneration and/or technical variability. dsCMA enables concurrent visualization of Aβ plaques and myelin microarchitecture at single-fiber resolution. Scale bar: 200 µm

To further evaluate the clinical utility of dsCMA, we analyzed cortical tissues from a cohort of four additional AD patients, two diabetic non-AD patients, and one non- neural disorder patient (Table 1; Fig. 10a–u). dsCMA resolved myelinated fibers at single-fiber resolution and revealed striking heterogeneity in both myelin organization and Aβ plaque distribution.

**Table 1:** List of human brain tissue specimens from AD patients

Patient	DX	Age	Sex	Notes
case #1	Hereditary Early onset Alzheimer’s Disease w/CVD	29	M	The patient demonstrated severe spastic paraparesis. Correspondingly, our microscopic examination of the brain revealed degeneration of the corticospinal tracts
case #2	Alzheimer’s disease	91	M	History significant for small bowel obstruction/small bowel ischemia, status post small bowel resection, atrial fibrillation
case #3	Alzheimer’s disease	63	M	History of cognitive decline over the past 5 years. He had expressive aphasia that reportedly started in 2013. Imaging studies showed moderate diffuse cerebral volume loss and small old lacunar infarcts in the right basal ganglia and left cerebellum. There was mild non-specific white matter disease likely representing chronic microvascular ischemic changes
case#4	Alzheimer’s disease	96	M	n/a
case#5	Alzheimer’s disease	70	F	4–5 year history of likely progressive supranuclear palsy with eye movement abnormalities, gait and balance impairment as well as speech and language impairment and unable to speak in recent years. She has a past history of Hashimoto encephalopathy, but the etiology is unclear on whether the diagnosis was specific
case#6	Alzheimer’s disease	83	M	n/a
case#7	Control	62	M	History of coronary artery disease, type II diabetes mellitus, and hypertension, hospitalized for st segment elevation myocardial infarction (stemi). the hospital course was complicated by septic shock, cardiogenic shock, acute renal failure, and acute hypoxemic respiratory failure
case#8	Control	69	M	History of pulmonary embolus on eliquis; end stage renal disease secondary to hypertension on hemodialysis, status post left renal transplant complicated by rejection and metastatic donor-derived neuroendocrine carcinoma, on chemotherapy who presented with myocardial infarction and heart failure
case#9	Control	73	F	History of diabetes mellitus and hypertension. Hypoxemic respiratory failure

Myelin variability manifested across four dimensions: (1) structural divergence in gyral/sulcal topology; (2) asymmetry in myelin orientation and morphology patterns across adjacent gyral regions; (3) morphological diversity, from coherent bundles to fragmented clusters, and gradients in fiber density from superficial to deep cortical layers; and (4) inter-sample staining variability, ranging from sparse "ghost" fibers to densely labeled tracts (Fig. 10c, f, i, l, o, r, u).

Aβ plaque burden also exhibited marked individual variation in AD cases, with difference in size, density and laminar distribution (Fig. 10 a, d, g, j). Notably, diabetic non-AD patients-intended as controls- also displayed Aβ plaque deposition (Fig. 10m, s), implicating diabetes in dysregulated Aβ metabolism. In contrast, the non-AD, non-diabetic control lacked plaques (Fig. 10q). Furthermore, all Aβ deposits positives cases (AD and diabetic non-AD) exhibited large, high-intensive Nissl+ staining blocks, some colocalizing with Aβ plaques, which disrupted baseline cytoarchitecture, producing a pathologically "dirty" parenchymal landscape (Fig. 10b, e, h, k, n, t, blue dashed circles).

Plaque-proximal regions showed microenvironment-specific effects: focal myelinated fibers degradation coexisted with structural preservation, even within the same patient, suggesting amyloid deposition may selectively compromise certain myelinated fibers’ integrity under permissive conditions (Fig. 10c, f, i, o, r, u, green squares).

We performed conventional MBP immunohistochemistry, it exhibited inconsistent performance across human samples (data not shown). These findings highlight the intricate interplay between Aβ pathology and myelin dynamics, compounded by systemic comorbidities such as diabetes, which may independently influence neuropathological outcomes.

## Discussion

### Methodological advancements related to dsCMA

AD research has shifted from a neuron-centric view [20, 21] to recognizing myelin as a critical component of disease pathology [22, 23]. However, existing myelin tools remain limited. Luxol Fast Blue has low signal-to-noise, whereas Gallyas silver and Black Gold can provide high-quality images but suffer from quality-control issues and restricted applicability across regions [24, 25]. Immunohistochemistry and myelin dyes depend heavily on tissue integrity and antibody performance and remain costly for large-scale use [26, 27]. Optical methods such as polarized light microscopy [28] and SCoRe imaging [17, 29] provide complementary perspectives but are constrained by resolution, orientation dependence, and processing demands [30–32]. Despite advances in animal models, examination of human tissue remains the gold standard for AD diagnosis [33]. MRI and PET have revealed important features of neurodegeneration but still lack sufficient resolution and require complex analysis pipelines [34, 35]. Together, these limitations—combined with the scarcity of human samples—underscore the urgent need for robust approaches that can maximize the value of both archival and newly collected specimens [36].

To address these gaps, we developed dsCMA, which integrates multiplex fluorescence and brightfield imaging to enable same-section analysis of cytoarchitecture, myeloarchitecture, and pathological markers, thereby facilitating detailed mapping of Aβ-associated myelin alterations. dsCMA is broadly compatible with existing staining workflows, although its lipid sensitivity limits use in paraffin-embedded tissues [37]. A major advantage of dsCMA is its enhanced sensitivity and stability in resolving gray matter myelinated fibers in both mouse and human tissue. By combining lipid-preserving fluorescence labeling (e.g., Nissl and Aβ) with lipid-dependent brightfield myelin contrast (myelRed), dsCMA enables sequential imaging of cytoarchitecture, plaques, and myelinated fibers from the same tissue section, minimizing registration errors inherent to multi-section comparisons (Figs. 3, 7, 8, 9).

Although SCoRe provides superior subcellular resolution, its orientation dependence, sensitivity to surface irregularities, and inability to reliably estimate myelin thickness often produce uneven backgrounds over large fields. Antibody-based myelin approaches are further limited by epitope instability, high background in archival tissues, and extensive optimization requirements. In contrast, dsCMA enables smooth serial imaging across low- and high-magnification scales, allowing efficient visualization of both global myelin architecture and plaque-associated microstructural disruption with high spatial continuity (Figs. 3, 7, 8, 9). Importantly, dsCMA performance is less affected by postmortem tissue integrity or antibody quality, making it particularly suitable for large clinical datasets and longitudinal neuropathological studies [38–40]. Compared to antibody-dependent methods, myelRed’s lipid-based chemistry provides robust signal even in over-fixed or poorly preserved tissues, consistent with its successful application to human archival samples more than a decade old (data not shown). Moreover, dsCMA myelin staining requires only ~ 1 h, compared to > 48 h for common immunofluorescence protocols (e.g., MBP, PLP), representing an ~ 40-fold improvement in throughput (Figs. 3, 4, 4, 5, 7, 8; Extended Data Fig. 5). This scalability is essential for systematic analysis of large brain bank collections.

Consistent with this utility, dsCMA recapitulates the topographic myelin patterns observed using MBP immunostaining, Gallyas, Black Gold, and Luxol Fast Blue across both mouse and human tissues, but with improved resolution and signal-to-noise ratio (Figs. 3, 4, 5, 7, 8, 9). Notably, dsCMA also resolves fine microstructural features, including individual fibers within bundles and gray matter myelination, which are critical for detailed morphological analysis (Figs. 3, 4, 5, 6, 7, 8; Extended Data Fig. 5).

Finally, we developed an associated quantification pipeline that integrates myelin and Aβ imaging to measure plaque-associated axonal changes (Fig. 6). This method extracts single-axon signals from myelin-labeled images and identifies plaque locations in Aβ-labeled images using a semi-automated workflow (Fig. 6c). Axonal density surrounding each plaque is then quantified using concentric bands of increasing radius (Sholl analysis) (Fig. 6d), enabling systematic assessment of spatial and temporal relationships between plaques and adjacent myelinated axons (Fig. 6e–j). As a proof-of-principle, we applied this approach across three age groups and three brain regions, establishing a scalable framework that can be readily adapted for quantitative analysis of human AD tissue.

### Our findings in 5xFAD mice

In this study, using the 5xFAD mouse model, we investigated the complex nature of neuritic plaques, which consist of a dense Aβ core surrounded by dystrophic neurites that may originate from axons, dendrites, or degenerating neurons, although the underlying mechanisms remain unclear [41–45]. Previous studies have reported demyelination associated with Aβ deposition in both transgenic models and human AD tissue [46–48], and ultrastructural evidence supports an axonal origin for at least a subset of dystrophic neurites [47, 49, 50].

Using dsCMA, we identified strong spatial associations among Aβ deposits, dystrophic neurites, and myelin defects, detectable as early as 2–3 months and progressively worsening with age. Quantitative analysis revealed a substantial decline in myelin integrity by 7 months in the cortex, dorsal hippocampus, and ventral hippocampus (e.g., ~ 30% reduction in the cortex) compared with 3-month-old mice (Fig. 6f–h). We further observed a significant negative correlation between regional plaque burden and the average axonal density surrounding individual plaques, with axonal density decreasing markedly with age (Fig. 6e). LAMP1 staining showed that dystrophic neurites, characterized by spheroids and swellings, were already associated with Aβ deposits by 3 months and frequently corresponded to regions of reduced or absent myelin. Notably, contrary to prior assumptions that dystrophic neurites are predominantly unmyelinated [46], we observed that a subset of dystrophic neurites remains myelinated, highlighting complex interactions between plaque pathology and myelin remodeling [51–53]. The proportion of myelinated dystrophic neurites increased from 3 to 13 months, suggesting a progressive degenerative process that may contribute to downstream mechanisms such as neuroinflammation [54].

Although we lack ultrastructural confirmation to directly quantify axon density, multiple observations support the interpretation that the detected changes reflect demyelination rather than stable developmental hypomyelination or primary axonal degeneration. First, fluorescent Nissl staining revealed preserved cellular architecture adjacent to plaques despite local myelin loss (Figs. 1, 2, 3; Extended Data Figs. 1, 2), suggesting that widespread neuronal loss is unlikely to fully explain the observed reductions in myelin. Second, myelin loss occurred in temporal association with progressive plaque accumulation (Fig. 6), inconsistent with stable developmental myelination patterns. Third, dsCMA revealed myelin fragmentation features, including irregular spheroids and staining blocks (Extended Data Fig. 5), which are not typical of normal unmyelinated axons. Finally, age-matched WT mice exhibited dense and continuous myelination in equivalent regions (data not shown), arguing against innate hypomyelination in plaque-associated zones.

We also detected early white matter damage by 3 months, with particularly prominent involvement of the ventral corpus callosum, a previously underappreciated phenotype closely associated with Aβ deposits. These findings further support the hypothesis that local lipid perturbations surrounding plaques and dystrophic neurites contribute to myelin instability, consistent with emerging evidence implicating lipid dysregulation in AD pathogenesis [55]. Notably, cortical plaques were associated with fewer dystrophic neurites than hippocampal plaques. Although absolute myelin loss in hippocampal regions may appear modest, even sparse myelination is critical for maintaining circuit integrity. For example, the alveus tract, a major hippocampal output pathway supporting hippocampal–cortical communication, showed plaque-associated myelin fragmentation (Fig. 5). Similarly, relatively sparse myelination in the perforant path plays a key role in regulating entorhinal–hippocampal coupling, and a ~ 30% local reduction in myelinated fibers could destabilize theta oscillations essential for memory encoding. Together, these changes may contribute to hippocampal–cortical network decoupling, impairing memory consolidation and spatial navigation and potentially accelerating cognitive decline.

In conclusion, the regional and temporal heterogeneity observed in the 5xFAD model highlights the need for further investigation into myelin vulnerability and its interaction with Aβ pathology across disease stages [7]. Our quantitative framework not only measures plaque burden and myelinated axonal density but also reveals dynamic spatial and temporal relationships between Aβ plaques and myelin degeneration across brain regions and ages (Fig. 6). This approach can be extended to large-scale studies of additional AD pathological markers beyond Aβ, enabling systematic characterization of circuit-level degeneration throughout disease progression in both animal models and human postmortem tissue.

### Our findings in human tissue

Consistent with our 5xFAD data, analysis of human AD tissues revealed complex and heterogeneous relationships between Aβ deposits and myelin integrity, challenging the prevailing assumption that Aβ deposits are inherently myelin-toxic [46, 56, 57]. Instead, our findings suggest that myelin defects in AD reflect not only local Aβ deposition but also regional brain architecture and selective axonal vulnerability [7, 22, 58–60]. Importantly, the impact of Aβ on myelin varies markedly across individuals, underscoring the need for caution when generalizing from limited datasets.

In a young familial AD patient (29 years old), Aβ deposits within white matter displayed an unusual elongated oval morphology aligned with myelinated fibers, a feature not previously reported [61, 62]. In gray matter, Aβ deposits also exhibited preferential alignment with local myelin trajectories, warranting further investigation. We further observed a progressive reduction in cortical parallel bundle length from gyri to sulci [63]. Notably, in this early-onset familial AD case, blood vessel-like structures were localized exclusively within gray matter, extending to the level of individual vessels.

Compared with the relatively uniform dense-core deposits observed in 5xFAD mice, human Aβ pathology exhibited striking heterogeneity in plaque morphology, size, and internal structure [64–66]. In a sporadic AD patient (91 years old), Aβ deposits showed region-specific distribution patterns across cortical and hippocampal areas and were variably associated with myelin defects, revealing previously unrecognized deposition phenotypes and myelin responses [67, 68].

A prominent feature of human hippocampal myeloarchitecture, distinct from mice, is a dense white matter band within the stratum radiatum (SR) and stratum lacunosum moleculare (SLM), which is not present in the mouse hippocampus [69, 70]. Within human hippocampal subfields, Aβ deposits were largely confined to gray matter and exhibited diverse interactions with myelinated fibers, ranging from minimal disruption to prominent myelin defects [71].

In addition, the human CA4/dentate gyrus (DG) region, which is essential for memory processing, is substantially expanded relative to mice and contains a complex network of myelinated fibers lacking clear dominant orientation or bundle organization [72]. Within CA4, Aβ deposits showed no clear subregional preference, suggesting relatively uniform vulnerability to Aβ pathology [73].

Finally, analysis of additional AD and non-AD cortical specimens confirmed that Aβ deposits are not consistently associated with myelin defects, supporting a more nuanced relationship between Aβ pathology and myelin integrity than previously recognized [7, 53, 72]. Together, these findings expand current understanding of human AD neuropathology by revealing region-dependent and patient-specific interactions between plaques and myelinated circuits [60, 73–76].

### Limitations of this study and future directions

In this study, we used 5xFAD mice, a widely adopted early-onset AD model, to investigate spatial correlations between myelin alterations and amyloid deposits, as well as other AD-related pathology. However, the 5xFAD model does not capture several critical aspects of AD, particularly tauopathy, which is strongly associated with cognitive decline [77, 78]. Therefore, extending dsCMA and the analytical framework established here to additional mouse models will be essential for a more comprehensive understanding of myelin–pathology interactions across disease progression.

We did not systematically examine whether sex influences Aβ-associated myelin vulnerability independently of plaque burden, which warrants future investigation. Moreover, it remains unresolved whether plaque-associated myelin loss reflects primary demyelination, secondary axonal degeneration, or compensatory regeneration. While our descriptive and quantitative findings establish dsCMA as a powerful platform, mechanistic dissection will require higher-resolution and longitudinal approaches, including electron microscopy (EM), in vivo imaging, combinatorial axon labeling, transgenic models, and AI-assisted quantification across ages and regions. These strategies will help distinguish axonal loss from demyelination and identify spatiotemporal “hotspots” of plaque-associated myelin disruption for mechanistic follow-up.

In addition, we did not assess molecular changes in myelin-associated proteins (e.g., PLP, CNPase) or nodal/paranodal markers (e.g., Caspr, Neurofascin). Such analyses would require multiplex immunostaining or proteomic approaches, which were beyond the scope of this study due to technical constraints, including spectral overlap with Aβ dyes and antibody compatibility.

Importantly, plaque progression differs between mice and humans: plaques appear first in the hippocampus in 5xFAD mice, whereas in humans they typically emerge first in neocortex and later in hippocampus [79]. Comparative studies across early-onset AD (EOAD), familial mutation-driven models (including 5xFAD), and late-onset AD (LOAD) will therefore be important for clarifying shared versus subtype-specific mechanisms. In the present study, we examined only two human brain specimens, and the limited sample size and heterogeneity preclude broad conclusions regarding Aβ–myelin interactions in humans. We also did not assess additional co-pathologies (e.g., TDP-43 or α-synuclein), which may independently influence myelin integrity and axonal pathology. Our goal was not to provide definitive comparative analyses, but rather to demonstrate dsCMA applicability to archival human tissue of variable quality (e.g., young familial AD versus aged sporadic AD) and to highlight shared features with 5xFAD mice while recognizing human-specific complexity, including gyral/sulcal plaque distribution, heterogeneous plaque morphologies, and region-dependent variation in myelin orientation and bundle length.

Despite these limitations, dsCMA enables high-resolution myelin visualization in postmortem human tissue without paraffin embedding and is less affected by postmortem degradation than conventional immunostaining. Its Aβ detection sensitivity and specificity were validated against Amylo-Glo, Thioflavin-S, and antibody-based assays. Applied to a broader cohort of human cortical samples, dsCMA revealed pronounced heterogeneity in Aβ–myelin relationships and highlighted major translational challenges, including the lack of standardized histological ground truth in human studies and the impact of systemic comorbidities (e.g., diabetes, hypertension, renal failure, cancer therapies, organ transplantation). These confounds complicate interpretation of mechanistic relationships between Aβ dynamics, myelin integrity, and systemic disease. Notably, dsCMA resolved myelin architecture in suboptimal specimens where MBP immunohistochemistry failed, underscoring its robustness for clinically heterogeneous human tissue. Nevertheless, definitive conclusions remain limited by biological variability and sample size, emphasizing the need for larger, clinically stratified cohorts.

We anticipate that dsCMA will be broadly adopted for large-scale postmortem studies of cytoarchitecture, myeloarchitecture, and pathological progression using brain bank resources worldwide. Given the rising global burden of neurodegenerative disease in aging populations, improved approaches are urgently needed to enable early prevention, accurate diagnosis, and effective interventions. Future work will extend dsCMA to investigate spatial and temporal relationships between myelin alterations and tauopathies, providing deeper insight into another central dimension of AD pathogenesis.

## Materials and methods

### Animals

The 5xFAD mouse transgenic model with co-expresses five human familial AD (FAD) mutations under the neuron-specific Thy1 promoter, characterized by the early age Abeta deposits (1.5–2 months), rapid and aggressive development of amyloid-beta (Aβ) pathology and associated cognitive and motor deficits. It recapitulates many key neuropathological features of human AD, including Aβ plaques, neuroinflammation, synaptic dysfunction, neuronal loss, and gender differences (female mice generally show higher level Abeta) and is widely used for Alzheimer’s disease (AD) research [80, 81]. Male and female 5xFAD transgenic AD mice, along with wild-type counterparts, aged postnatally at 2–3, 7, and 12 and older months, were procured from Jackson Laboratories with mixed C57BL/6 and SJL background (https://www.jax.org/strain/006554). These animals were housed at the UCLA vivariums, with unfettered access to food and water. Living conditions were regulated for temperature (21–22 °C), humidity (40–51%), and light (12-h light/dark cycle). All protocols adhered to the ethical guidelines set by the Institutional Animal Care and Use Committee (IACUC) of the University of California, Los Angeles.

*Human AD brain tissue:* Fixed brain tissue specimens from AD patients were sourced from the Department of Pathology and Laboratory Medicine at the David Geffen School of Medicine, UCLA (DGSOM). Processing of these tissues followed protocols analogous to those used for mouse specimens.

### Tissue collection and sectioning

The established protocols for animal brain extraction, histology, and imaging have been previously described. Briefly, subjects were deeply anesthetized with sodium pentobarbital (Euthasol, 2 mg kg^−1^, intraperitoneally), followed by transcardial perfusion with saline and 4% paraformaldehyde in 0.01 M PBS. After overnight post-fixation, brains were embedded in 4% agarose and sectioned at 25 or 50 µm using a vibratome (Leica VT1000 or Compresstome 200). Sections were stored in 0.01 M PBS, mounted on hydrophilic plus slides (Bio SB), and stored at 4 °C for short-term or in antifreeze medium (40% glycerol, 60% 0.01 M PBS) at − 20 °C for long-term preservation [82].

### Double scan cyto-myelo-architecture assay (dsCMA) pipeline

#### First round—multiple fluorescent staining

Sections underwent following staining for different purposes, staining with lipid detergent treatment for multiplex fluorescent imaging, without lipid detergent treatment for following myelRed myelin staining:

1. The comparison between amyloid plaques with Amylo-glo (AmG, Histo-Chem Inc) and Thioflavin-S(ThioS, Millipore Sigma, cat no. T1892-25G). Sections were mounted on hydrophilic plus slides ( BioSB, cat no. BSB 7028), air dried at room temperature, incubated in 1 × Amylo-Glo in 0.9% saline for 20 min, slides were rinsed in 0.9% saline, temporarily sealed with saline, imaging under fluorescent model. After imaging, coverslips were removed, slides were rinsed very briefly in ddH2O, then incubated 1% (ddH2O) Thioflavin-S solution for 10 min, dehydrated in serial ethanol solution, sealed with 40% glycerol (0.01 M PBS) and rescan. The AmG and ThioS staining patterns were registered using Warpy process [16] (https://imagej.net/plugins/bdv/warpy/warpy) and the overlapped Abeta plaques were calculated.
2. The spatial relationship of amyloid plaques, myelin and dystrophic neurites: optional staining combination: Myelin basic protein (MBP) plus AmG; 2′,3′-cyclic nucleotide 3′-phosphodiesterase (CNPase) plus AmG; lysosomal-associated membrane protein 1(LAMP1) plus presenilin-1(PSEN1) plus APP/Abeta; APP/Abeta plus AmG. Briefly, sections were incubated in blocked solution (1% donkey serum, 0.3% Triton X100, 0.01 M PBS) for 2 h, primary antibodies diluted in block solution and incubated for 48 h at 4 degree, after washed 3 times each time for 15 min in 0.01 M PBS, second antibodies were diluted in blocked solution and incubated for 2 h at room temperature, after washed 3 time each time 15 min in 0.01 M PBS, slides were sealed by 40% glycerol in 0.01 M PBS, and ready for imaging.

Following list antibodies information:

**Table:** 

1st antibody	Vendor	cat ID	Dilution
Phospho-Tau	Thermo fisher science	MN1020	1:300
MBP	Abcam	ab218011	1:500
CNPase	Encor	RPCA-CNP	1:1000
LAMP1	Dshb	1D4B	1:300
PSEN1	Thermo fisher science	PA5-96088	1:200
App/Abeta	Cell signaling technology	2450 s	1:500

All 2nd antibodies specific target 1st antibodies obtained from Jackson ImmunoRes, diluted 1:500.

3. Fluorescent Nissl staining was used instead of nuclear counterstaining (e.g., DAPI) to provide detailed visualization of somatic structure and cytoarchitecture, offering superior anatomical context for assessing myelin and Aβ plaque morphology. Amyloid plaques stained by Amylo-glo followed with fluorescent Nissl staining to show brain cytoarchitecture: Aired dried sections mounted slides were incubated in NeuroTrace 640/660 (1:500 in 0.01 M PBS, Thermofisher cat no. N21483) for 3 h at room temperature, washed 3 times each for 5 min, then incubated in 1 × Amylo-Glo in 0.9% saline for 20 min, slides were rinsed in 3 times each time 5 min in 0.01 M PBS, sealed in 0.01 M PBS for confocal or fluorescent microscope imaging.
4. SCoRE was performed using a Leica SP8 confocal microscope set in reflection mode at 551 nm at the Advanced Light Microscopy/Spectroscopy Laboratory and Leica Microsystems Center of Excellence at the California NanoSystems Institute at UCLA (RRID:SCR_022789) with funding support from NIH Shared Instrumentation Grant S10OD025017 and NSF Major Research Instrumentation grant CHE-0722519″

*Second round—myelred staining:* After first round imaging, slides coverslips were removed, sections were briefly washed 3 times in 0.01 M PBS. A fresh staining solution was prepared immediately before use by combining chloroauric acid and trisodium phosphate at a 1.4:4.2 ratio, followed by a 1:7 dilution in distilled water. The staining solution was pre-warmed to 55 °C and equilibrated for ≥ 15 min. Mounted sections were directly immersed in the staining solution and incubated for 15–20 min at 55 °C. The staining reaction was stopped by transferring slides to 0.9% NaCl at room temperature. Staining time may require optimization depending on tissue thickness and fixation conditions. Following staining, slides were dehydrated through a graded ethanol series (50%, 70%, 80%, 90% for 1 min each; 95% × 2; 100% × 2, 1 min each), cleared in xylene (2 × 1 min), and coverslipped using DPX mounting medium. All steps were performed with gentle agitation to promote even penetration of the dye and prevent precipitate formation.

### Section imaging

Fluorescent and brightfield images were acquired using an Olympus VS120 or VS200 epifluorescence microscope equipped with 10 × or 20 × Plan Apochromat objectives (NA 0.40–0.75), depending on experimental requirements, whereas conventional confocal imaging was acquired by Dragonfly confocal microscope with a 40 × water-immersion objective (NA 1.1). Selected high-resolution images of amyloid plaques and labeling-free myelin were acquired using spectral confocal reflectance microscopy (SCoRe) with a 40x (NA 1.1) Leica confocal lens or STED microscope at CNSI, UCLA to achieve high axial resolution for fine myelin structure analysis. Captured multichannel photomicrographs were processed to delineate amyloid plaques (Aβ)and Nissl staining cytoarchitecture, FITC channel was used for myelin fluorescent imaging.

### Co-registration of different model images

The Warpy method and plugin was employed for co-registration of fluorescent and brightfield images from the same section [16] according to the online settings and protocol instruction (https://imagej.net/plugins/bdv/warpy/warpy). To achieve accurate inter-modal alignment, image registration was carried out using a combined Fiji/BigWarp and QuPath workflow. Images were converted to TIFF or BTF formats with an accurate physical scale, imported into QuPath (version, v 0.3 +) projects linked to both fluorescence and MyelRed image datasets. The QuPath project was then opened in Fiji ImageJ (version, 1.54j) using the Warpy plugin for semi-manual rigid registration (https://imagej.net/plugins/bdv/warpy/warpy), and refined manually using BigWarp to ensure anatomically correct landmark correspondence. The fluorescence channel was designated as the fixed reference image, while the MyelRed image served as the moving image. Local adjustments were applied as necessary to correct regional distortion and tissue curvature. The final transformation parameters were exported as a JSON file and stored within the QuPath project directory. QuPath subsequently used this transformation to transfer all PathObjects (including manual annotations and automated detections) bidirectionally between the two image modalities with spatial fidelity.

### Data analysis and statistics

Fiji ImageJ, QuPath and BIOP Warpy pipeline facilitated 2D Abeta deposits and myelin image registration, segmentation and statistical analysis [16]. 3D Abeta deposits, myelin and dystrophic neurites interaction, and manually single fiber tracking were analyzed by MIP screening and virtual slicing functions in Imaris. Figures were prepared by Biorender.

*2D quantification analysis:* We developed a novel in-house method to quantitatively analyze amyloid plaques and their interactions with myelinated axons. In brief, four representative hemisphere images were collected from four 5xFAD male and female animals per age group (three in the case of the 3-month-old group). Individual plaque locations within each image were identified using a laplacian of Gaussian (LoG) filter, which detects blob-like structures [83]. The plaque coordinates were then exported as an SWC file [84] and manually edited in Vaa3D to generate the final set of plaque coordinates within the three selected regions: neocortex (CTX), dorsal hippocampus (dorsal HPF), and ventral hippocampus (ventral HPF). We then compared plaque counts across these regions and age groups. We carried out pair-wise statistical comparison where each region was compared directly to the other two regions (a total of three pair-wise comparisons). Similarly, each age group was compared with the other two age groups. For the pairwise comparisons, we used the nonparametric Wilcoxon Rank Sum test (*n* = 4 in all groups). Specifically, each age group included four animals (3-month group: 3 males and 1 female; 7-month group: 2 males and 2 females; 12-month group: 2 males and 2 females). This sample size was not sufficient to support a statistically powered analysis of sex-dependent effects, and we therefore did not attempt formal comparisons between males and females.

To assess axonal density surrounding each plaque, we adopted a Sholl-like analysis [85], a method widely used in single-neuron morphological quantifications. Myelinated single axons were identified by applying a Frangi filter [86] to the myelin signal. Axonal pixels surrounding each plaque were binned based on their distances from the plaque center in 20 µm intervals, effectively segmenting the surrounding area into concentric Sholl bands of increasing radii.

Each Sholl band is defined by two consecutive concentric circles, where the outer limit of each band extends 20 µm beyond the preceding circle. Axonal density within each band was quantified by dividing the total myelinated pixels within the band by the band’s area. This measurement was performed for each identified plaque, allowing for comparisons of plaque-surrounding axonal density across regions and age groups. For the plaque level axonal density analysis, we used the parametric Student’s t-test to compare the average axonal density of each region against the other two regions and each age group against the remaining two age groups. Finally, we pulled all age group data together to carry out overall region-based comparison, and then pulled all regional data together to quantify overall age-based comparison.

*3D quantification analysis:* Imaris, each Abeta deposit and surrounding MBP and LAMP1 signals were manually calculated, intact myelinated fibers penetrated through Abeta deposits were counted as positive events, total number and percentage were calculated.

## Supplementary Information

Below is the link to the electronic supplementary material.

Supplementary Material 1.

## Data Availability

No datasets were generated or analysed during the current study.
