# Supplementary material for: Investigation of the myelin-amyloid interplay in Alzheimer’s disease: insights from novel dsCMA imaging in mouse and human brains
Source: Acta Neuropathol Commun. 2026 May 14;14:145. doi: 10.1186/s40478-026-02317-8 (PMC13348811; doi:10.1186/s40478-026-02317-8)
Supplement: Supplementary file 1 — Supplementary Material 1. [file 40478_2026_2317_MOESM1_ESM.docx]

**Extended Data Figure legend:**

**
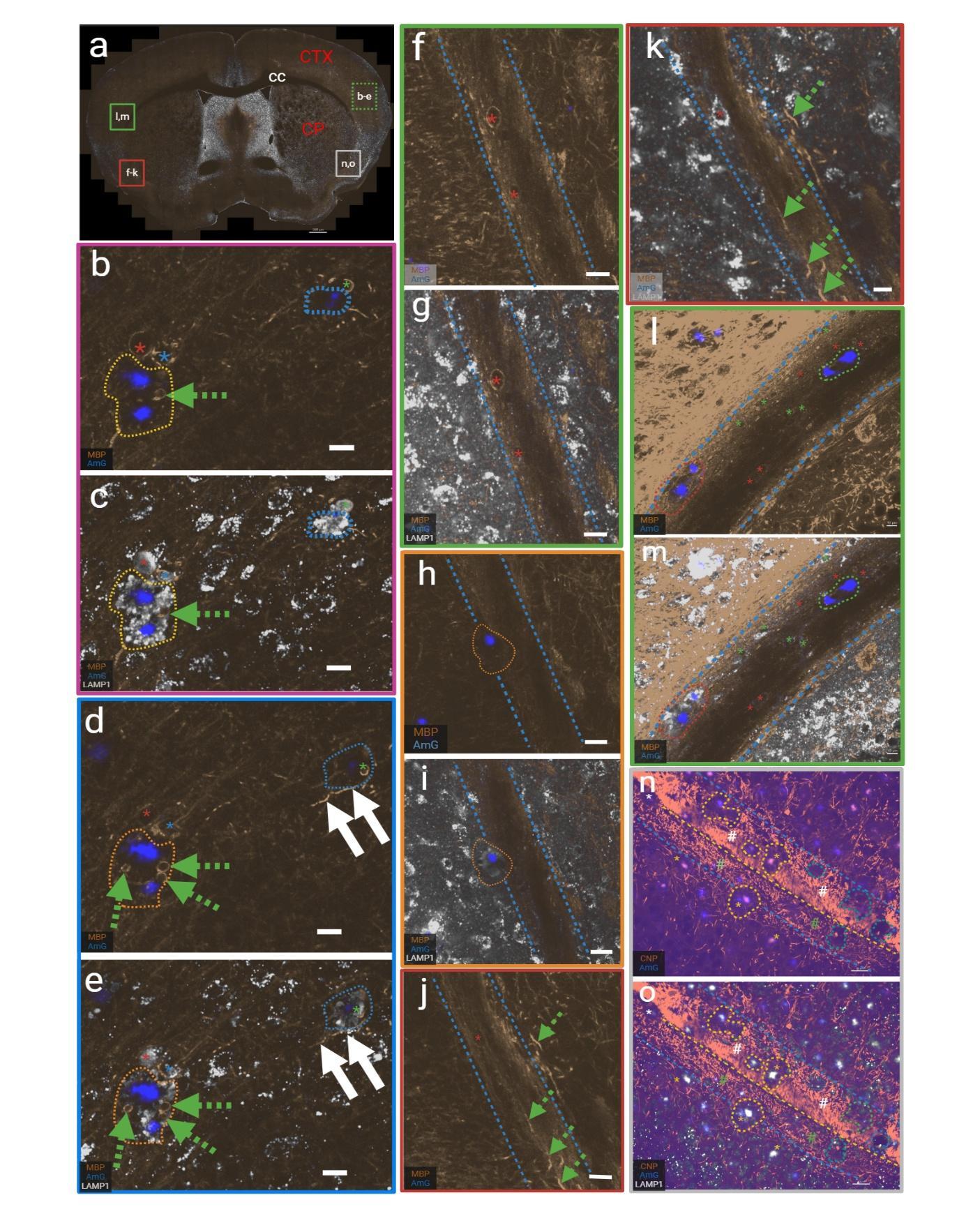
**

**Extended Data Figure 1 (related to Fig. 2). Diverse presentations of single-fiber and bundled myelin defects associated with amyloid-β (Aβ) deposits in gray and white matter of 5xFAD mice at 3 months (a–m) and 7 months (n, o).**

**a** Overview showing the distribution of Aβ (blue), LAMP1 (white; pseudo-colored), and MBP (orange; pseudo-colored) in a 3-month-old 5xFAD mouse brain. Scale bar: 500 µm.

**b, c** Confocal virtual slices showing LAMP1+ large dystrophic bulbs (*). A red star indicates a thin continuous MBP signal encircling a large LAMP1 bulb with heterogeneous intensity. A blue star marks an irregular region where MBP encapsulates a bulb with dot-like LAMP1 signals. A green star highlights a bulb partially encased by myelin. Green arrows indicate a bulb lacking detectable LAMP1 signal. Yellow and blue circles denote an Aβ deposit and adjacent small dystrophic neurites, respectively. Scale bar: 15 µm.

**d, e** Virtual slices of the same region showing newly formed LAMP1-negative dystrophic bulbs (green arrows). White arrows indicate disrupted myelin signal. Yellow and blue circles indicate the same Aβ deposit surrounded by dystrophic neurites, with variable LAMP1 and MBP signal intensities. Virtual slice distance from **b, c**: 6 µm. Scale bar: 15 µm.

**f, g** The blue dashed line marks the white matter boundary of the corpus callosum (cc). Red asterisks indicate myelin bulbs of varying size within white matter lacking LAMP1 signal. In gray matter, LAMP1+ dystrophic neurites surround Aβ deposits (blue). Scale bar: 15 µm.

**h, i** The blue dashed line delineates the white matter boundary of the corpus callosum (cc). An Aβ deposit at the white matter edge (orange circle) is associated with LAMP1 signal, without detectable co-localized myelin signal in either white or gray matter. Scale bar: 20 µm.

**j, k** The blue dashed line marks the white matter boundary of the corpus callosum (cc). A red star indicates LAMP1-associated myelin damage within white matter in the absence of detectable Aβ deposits. Green arrows indicate abnormal increases in myelin signal in both white and gray matter. Scale bar: 10 µm.

**l, m** The blue dashed line marks the white matter boundary of the corpus callosum (cc). Red stars indicate myelin damage in white matter without associated Aβ or LAMP1 signals. Green stars indicate myelin damage associated with LAMP1 signal but lacking Aβ. A red circle highlights myelin damage colocalized with both Aβ and LAMP1 signals, whereas a green circle indicates Aβ signal without myelin damage or LAMP1 signal. Scale bar: 10 µm.

**n, o** Representative images from 7-month-old 5xFAD mice showing white matter myelin damage labeled by CNPase (deep orange; pseudo-colored). The blue dashed line marks the corpus callosum (cc) boundary, and the yellow line delineates the alveus (alv; white #) and external capsule (ec; green #). A green circle indicates myelin defects within the alveus without detectable Aβ or LAMP1 signal. A yellow circle indicates myelin damage associated with Aβ and LAMP1 signals. A white star marks myelin damage at the border between alveus and external capsule. A yellow star indicates residual myelin signal colocalized with LAMP1 and Aβ within the damaged region. Scale bar: 10 µm.

**
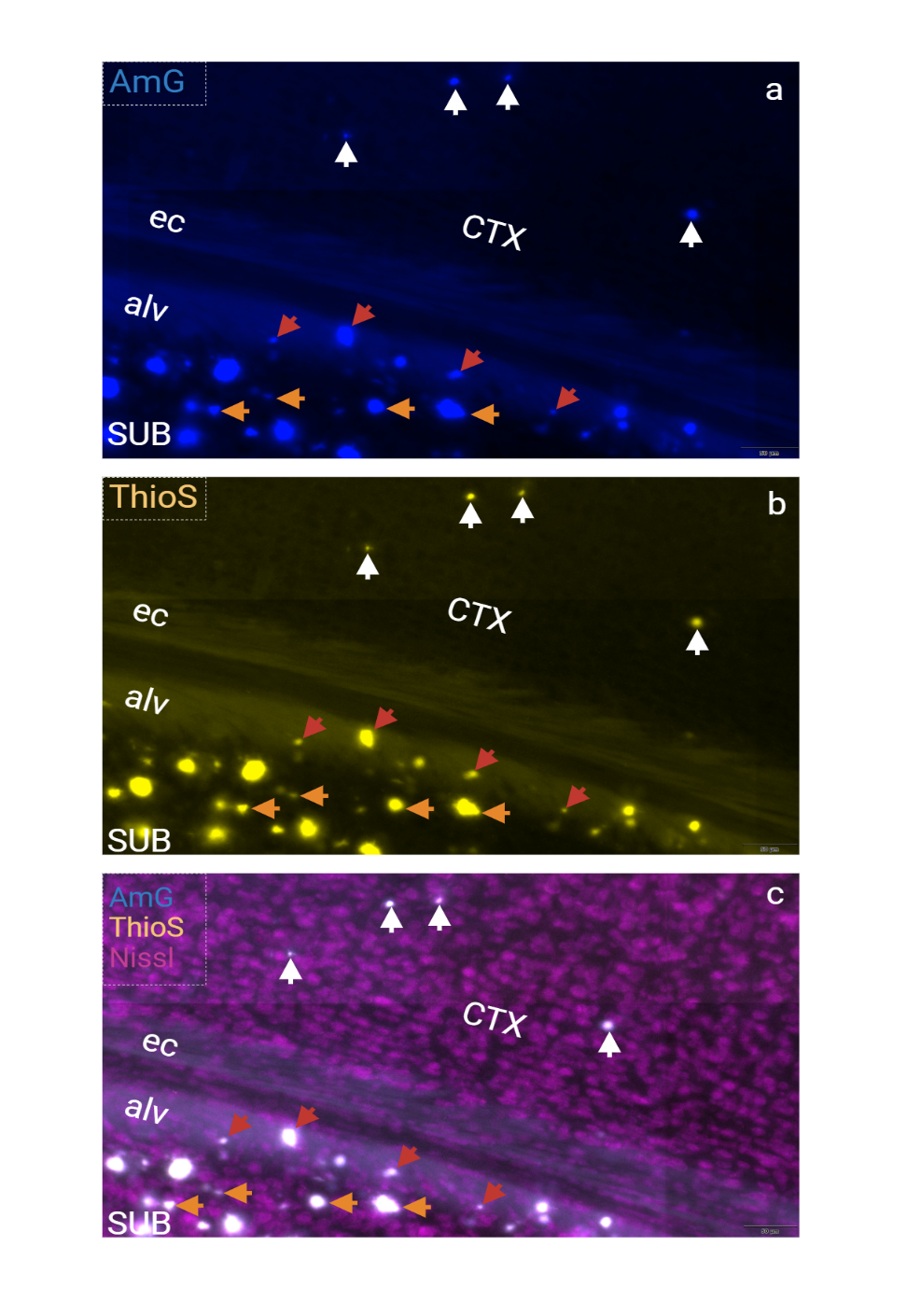
**

**Extended Data Figure 2 (related to Fig. 2). Amylo-Glo and Thioflavin-S reveal comparable Aβ deposit morphologies and distribution in a 3-month-old 5xFAD female mouse.** **a** Amylo-Glo staining shows Aβ deposits of varying size and morphology in cortical gray matter (white arrows), the white matter alveus (red arrows), and the hippocampal subiculum (yellow arrows).
 **b** Thioflavin-S staining (pseudo-colored) labels Aβ deposits with similar size and morphology in the same regions (white, red, and yellow arrows).
 **c** Overlay of **a** and **b** demonstrates >99% overlap between Amylo-Glo+ and Thio-S+ Aβ deposits, with no detectable differences in staining patterns. Scale bar: 50 µm (thickened for clarity).

**
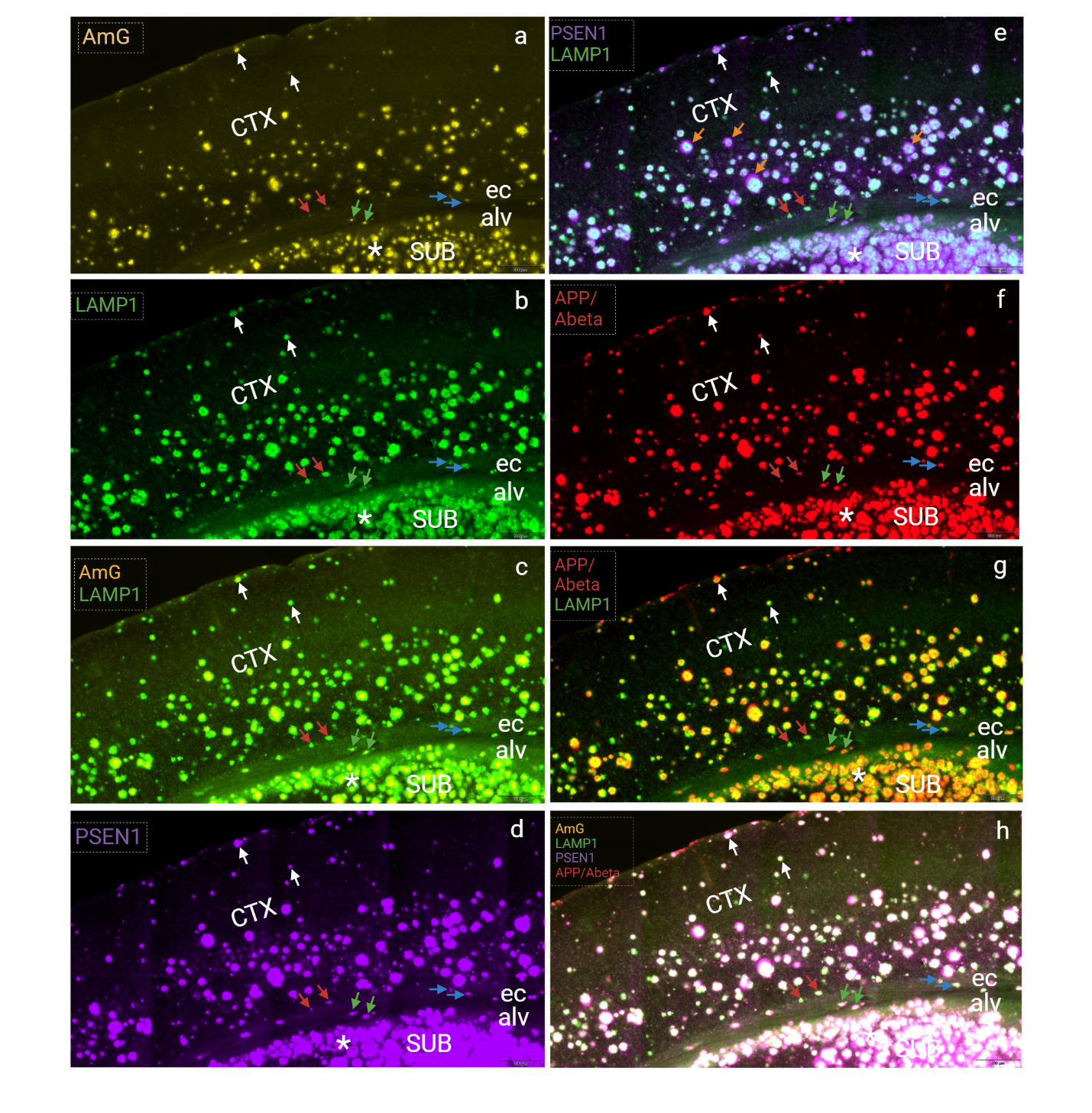
**

**Extended Data Figure 3 (related to Fig. 2). Amylo-Glo and immunolabeling reveal Aβ deposits, dystrophic neurites, and PSEN1 localization in a 7-month-old male 5xFAD mouse.** Scale bar: 200 µm.

**a** Amylo-Glo–labeled Aβ deposits (pseudo-colored) of diverse sizes and morphologies are distributed across cortical layers (white arrows), at the gray–white matter interface (red arrows), within the corpus callosum as dense-core plaques (green arrows) or plaques with blurred profiles (blue arrows), and at high density in the alveus and subiculum, where individual deposits are difficult to resolve (white star).

**b** LAMP1 immunostaining in the same region reveals dystrophic neurites associated with all Aβ deposit locations (arrows).

**c** Overlay of **a** and **b** shows complete colocalization of Amylo-Glo+ Aβ deposits with LAMP1+ signals, with LAMP1 forming flower-like structures around plaques.

**d** PSEN1 immunostaining in the same region shows PSEN1 signals localized to all Amylo-Glo+ Aβ deposit sites (arrows).

**e** Overlay of PSEN1 and LAMP1 demonstrates complete colocalization at plaque sites (arrows); at some locations, PSEN1 signals extend beyond LAMP1 labeling (yellow arrows).

**f, g** APP/Aβ labeling using NAB228 shows that all Amylo-Glo+ Aβ deposits are NAB228-positive (arrows) and display a broader distribution; APP/Aβ signals also colocalize with LAMP1 (**g**).

**h** Composite overlay of Amylo-Glo, LAMP1, PSEN1, and APP/Aβ signals illustrates their spatial relationships and co-labeling at Aβ deposit sites.


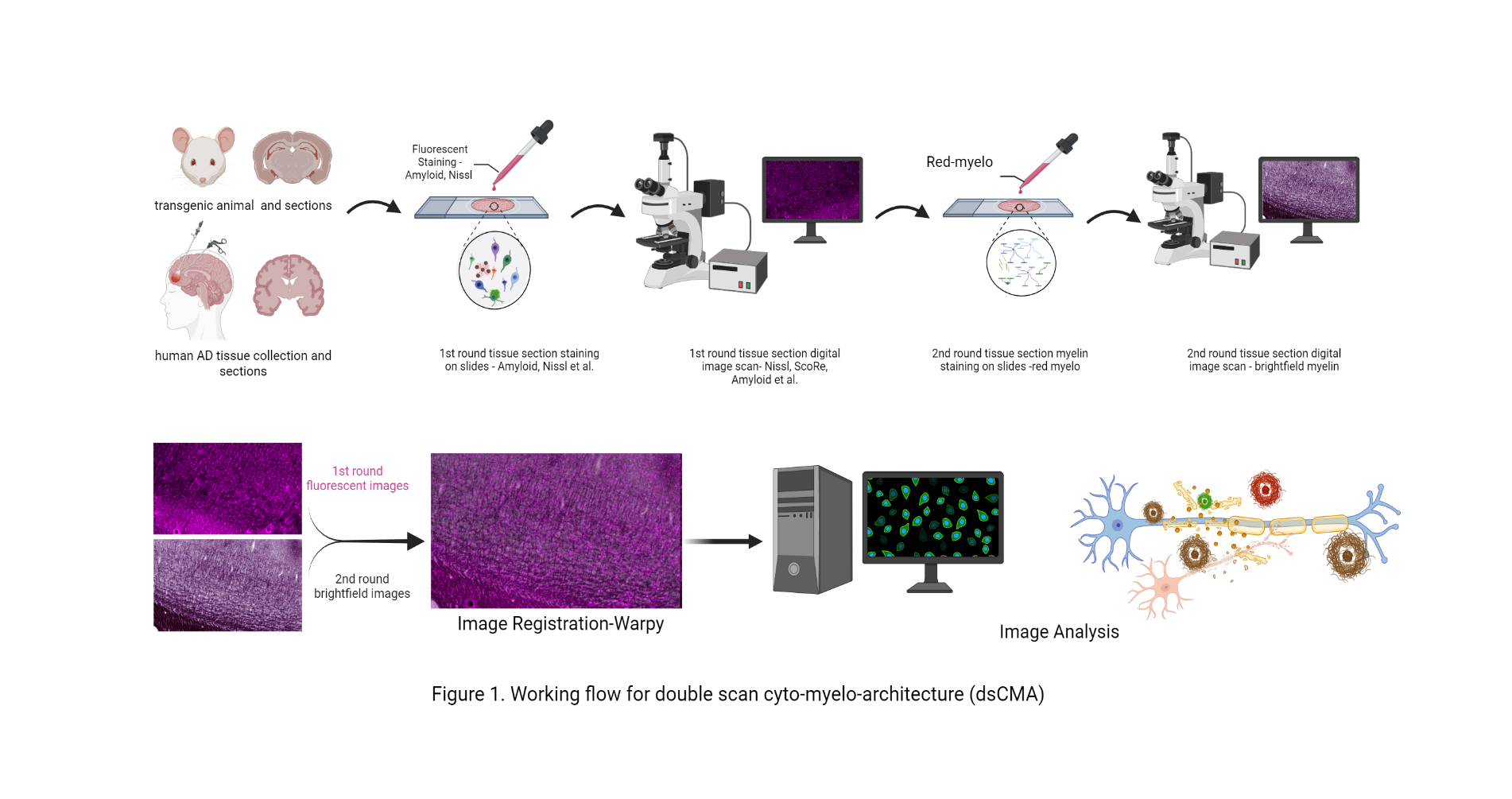


**Extended Data Figure 4. Schematic representation of the double scan cyto-myelo assay (dsCMA) workflow and pipeline.**


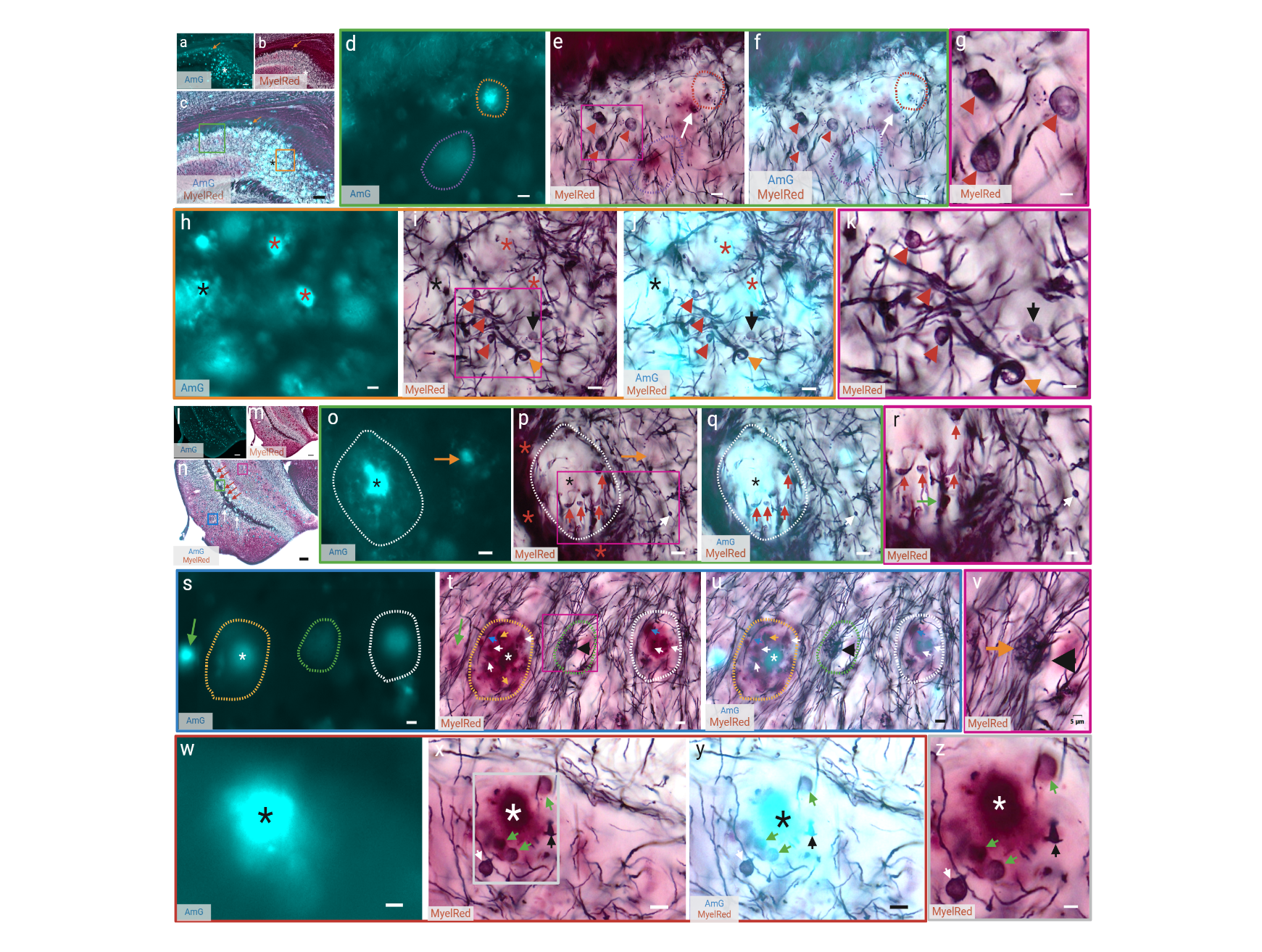


**Extended Data Figure 5 (related to Fig. 5). dsCMA pipeline reveals diverse myelin fiber morphologies and plaque-associated abnormalities in a 12-month-old 5xFAD mouse.** **a–c** Overview of dorsal hippocampal/subicular region. **a** Dense Aβ deposits are prominent in the subiculum (white star). **b** Corresponding myeloarchitecture. **c** Co-registration of **a** and **b** shows a white matter defect colocalized with an Aβ deposit (orange arrow) and short disrupted myelinated fiber segments forming a distorted network (black star). Scale bar: 250 µm.
 **d–g** Enlarged view of the green box in **c**. **d** Aβ deposits include a small dense-core plaque with thin halo (orange dashed circle) and a diffuse halo plaque lacking a core (purple dashed circle). **e** Corresponding myeloarchitecture. **f** Co-registration shows spheroids with clear (red arrowheads) or blurred (white arrow) profiles that are not associated with adjacent Aβ deposits (red dashed circle), whereas red staining blocks colocalize with the diffuse Aβ deposit together with normal-appearing fibers (purple dashed circle). **g** Higher magnification of the purple box in **e** highlights spheroids with distinct staining intensities and morphologies. Scale bars: **d–f**, 10 µm; **g**, 5 µm.
 **h–k** Enlarged view of the orange box in **c**. **h** Aβ deposits include single dense-core plaques without halo (red stars) and plaques with multiple grain-like cores and a large halo (black star), along with additional blurred deposits. **i** Corresponding myeloarchitecture. **j** Co-registration shows low-density but otherwise normal-appearing fibers at plaque locations (red and black stars), and multiple spheroids located between plaques with clear (red arrowheads) or blurred profiles (black arrow). **k** Higher magnification of the purple box in **i** shows spheroids with faint blurred profiles (black arrow), internal vacuoles (orange arrowhead), and clear profiles (red arrowheads). Scale bars: **h–j**, 10 µm; **k**, 5 µm.
 **l–n** Overview of ventral hippocampal/cortical region. **l** Abundant Aβ deposits are distributed throughout ventral cortex and hippocampus. **m** Corresponding myeloarchitecture. **n** Co-registration highlights widespread myelin defects within the external capsule (red arrows). Scale bar: 250 µm.
 **o–r** Enlarged view of the green box in **n**. **o** A large plaque containing a dense core with multiple grain-like cores and a large halo (black star; dashed white circle) coexists with a smaller dense-core halo plaque (orange arrow). **p** Corresponding myeloarchitecture. **q** Co-registration shows a large white matter defect (red stars) colocalized with the plaque (white dashed circle), accompanied by multiple spheroids (red arrows) and absent or weak staining blocks (black star; orange arrow). A small spheroid on a thin normal fiber is present without detectable Aβ (white arrow). **r** Higher magnification of the purple box in **p** shows multiple blurred-profile spheroids around the plaque (red arrows), including a compact spheroid cluster (green arrow) and a small spheroid on a thin fiber (white arrow). Scale bars: **o–q**, 10 µm; **r**, 5 µm.
 **s–v** Enlarged view of the blue box in **n**. **s** Aβ deposits exhibit multiple morphologies, including dense-core halo plaques (green arrow), blurred-core plaques with large halo (yellow dashed circle), diffuse faint halo plaques (green dashed circle), and large blurred-core plaques lacking halo (white dashed circle). **t** Corresponding myeloarchitecture. **u** Co-registration shows myelin debris staining blocks (white arrows) colocalized with plaque cores (white star) and halos (yellow dashed circle), intermingled with normal fibers (blue arrow) or degenerating residual fibers (yellow arrows). No spheroid-like degeneration is detected at the dense-core halo plaque (green arrow). A large spheroid with intricate substructures (black triangle arrowhead) is associated with a normal fiber and colocalized with a halo-only plaque (green dashed circle). **v** Higher magnification of the purple box in **t** highlights vesicle-like substructures within the spheroid (yellow arrow) and its associated myelinated fiber (black arrow). Scale bars: **s–u**, 10 µm; **v**, 5 µm.
 **w–z** Enlarged view of the red box in **n**. **w** Large dense-core halo Aβ deposit (black star). **x** Corresponding myeloarchitecture. **y** Co-registration shows a large staining block (white star) colocalized with spheroids exhibiting blurred (green arrows) and clear profiles (white arrow). A partially blurred spheroid associated with a myelin fragment is indicated (black arrow). **z** Enlarged view of the gray box in **x** shows a central staining block (white star) surrounded by multiple peripheral blurred-profile spheroid-like staining blocks (green arrows) overlapping the plaque position, including partial blurred (black arrow) and clear (white arrow) spheroids and multiple small dark debris-like spots. Scale bars: **w–y**, 10 µm; **z**, 5 µm.


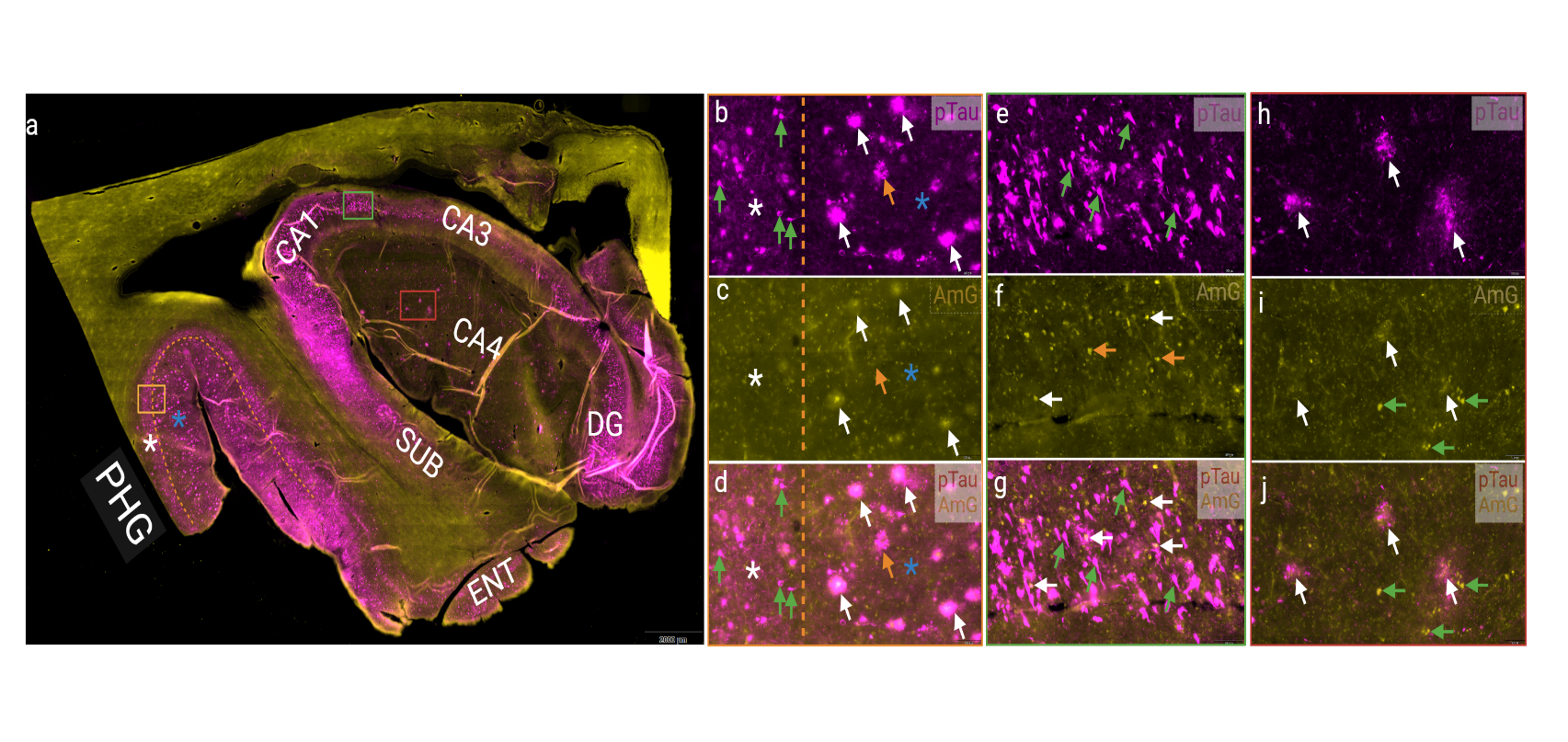
**Extended Data Figure 6 (related to Fig. 9). pTau staining patterns in a sporadic AD patient.**

**a** Overview of AT8 immunostaining showing pTau signals (purple) across the hippocampal formation, including PHG, ENT, SUB, CA1, CA3, and DG, with region-dependent densities. In PHG, a deep layer (white star) contains dense fibers intermingled with AT8+ cells, whereas the superficial layer (blue star) shows relatively sparse AT8+ cells and plaque-like AT8+ structures. Scale bar: 2000 µm.

**b** Enlarged view of the yellow box in **a** (PHG). Numerous small, dense AT8+ profiles consistent with degenerating neurons are present in the deep layer (white star; green arrows), whereas large AT8+ clusters are observed in the superficial layer (blue star; white arrows), including structures with complex internal features (yellow arrow).

**c** Corresponding Aβ labeling in the same region shows multiple AmG+ Aβ deposits with dense cores and halos at AT8+ cluster sites (white arrows), whereas one AT8+ cluster lacks detectable AmG signal (yellow arrow). AT8+ degenerating cell profiles in the deep layer show no associated AmG signal.

**d** Overlay of **b** and **c** showing that AT8+ signals may colocalize with AmG+ deposits (white arrow) or occur independently (yellow arrow).

**e** Enlarged view of the green box in **a** (CA1). Multiple AT8+ profiles consistent with degenerating neurons are observed (green arrows), without large AT8+ clusters.

**f** Corresponding Aβ labeling shows multiple small AmG+ spots lacking halos (white and yellow arrows).

**g** Overlay of **e** and **f** showing that AT8+ degenerating neurons do not overlap with AmG+ spots (white arrows), whereas degraded-like AT8+ signals colocalize with AmG+ spots (yellow arrows).

**h** Enlarged view of the red box in **a** (CA4). Multiple AT8+ signal clusters lacking clear neuronal profiles are present (white arrows).

**i** Corresponding Aβ labeling shows multiple small AmG+ spots without halos (green arrows) and weak, blurred AmG staining at AT8+ cluster locations (white arrows).

**j** Overlay of **h** and **i** showing overlap between degraded-like AT8+ signals and weak AmG staining (white arrows), whereas AT8+ signals do not colocalize with AmG+ spots (green arrows).
